# Artificial intelligence-driven diabetic retinopathy research: mapping the evolution, coupling, and global collaboration landscape (1996-2026)

**DOI:** 10.3389/fendo.2026.1935040

**Published:** 2026-09-10

**Authors:** Yihui He, Danyu Li, Danbing Li, Yunci Ma, Wentao Huang

**Affiliations:** 1 The Fifth Affliated Hospital, Southern Medical University, Guangzhou, Guangdong, China; 2 School of Computing and Artificial Intelligence, Guangzhou Xinhua University, Guangzhou, Guangdong, China; 3 School of Computer and Information Engineering, Xiangyang Vocational College of Science And Technology, Xiangyang, Hubei, China

**Keywords:** artificial intelligence, bibliometrics, deep learning, diabetic retinopathy, knowledge graph, machine learning, multimodal fusion

## Abstract

**Background:**

*Diabetic Retinopathy* (DR) is a major global cause of blindness. *Artificial Intelligence* (AI) has markedly impacted fundus screening over the past three decades, yet systematic bibliometric mapping of the knowledge architecture, evolutionary paths, and synergy among AI models, data modalities, and DR remains scarce.

**Objective:**

This study conducted a comprehensive bibliometric analysis to characterize the AI-driven DR knowledge structure, collaboration networks, and hotspot migration, and to elucidate co-evolutionary dynamics among AI advances, data modality development, and DR research.

**Methods:**

Following a systematic search and screening process, 12,741 publications were identified from the *Web of Science Core Collection* (WoSCC), PubMed, and Scopus, spanning from January 1996 to June 2026. The analytical framework combined multiple methodologies: VOSviewer for collaborative network visualization, CiteSpace for burst detection and timeline mapping, and a Python-based text-mining pipeline for standardized extraction and normalization of AI model names, data modalities, and disease entities.

**Results:**

The field exhibits a distinct three-stage evolutionary trajectory: the traditional machine learning era (1996-2014), the deep learning surge (2015-2019), and the current phase marked by the growing prominence of Transformer-based models (2020-present). The collaboration landscape is multipolar, with the United States, China, and India as hubs, while Singapore produces high-impact research. The knowledge base rests on algorithmic innovation and clinical validation. Convolutional neural networks have long served as the backbone architecture in the literature, while Vision Transformers have shown a clear upward trend in publication volume in recent years. Research hotspots are expanding from single-disease classification toward multimodal integration. Although fundus imaging remains the predominant data source, the potential of electronic health record narratives and multi-omics data is increasingly recognized. Overall, the research focus is shifting from “black-box” pattern recognition toward explainable AI and end-to-end clinical translation.

**Conclusion:**

This study presents a systematic bibliometric mapping of AI-driven DR research, revealing high-frequency co-occurrence patterns between architectural specialization and clinical demands. Challenges persist in data integration, rare-disease evidence, and cross-setting validation. The future is likely to be shaped by multimodal foundation models and portable acquisition, transitioning AI toward comprehensive clinical decision support.

## 1 Introduction

*Diabetic Retinopathy* (DR) is one of the most common microvascular complications of diabetes (1). Its prevalence has risen sharply alongside the global diabetes epidemic, imposing an ever-increasing burden on healthcare systems worldwide (2). Traditional diagnostic workflows rely on manual grading of color fundus photographs by trained ophthalmic professionals – an approach increasingly strained by specialist shortages and the uneven geographic distribution of ophthalmic resources (3, 4). This clinical urgency has catalyzed a paradigm shift toward *Artificial Intelligence* (AI) for automated, scalable, and objective DR screening and management. In parallel, advances in AI – with their powerful capabilities in image processing and pattern recognition – have opened new avenues for overcoming resource bottlenecks in DR screening (5). AI-driven DR research has progressively evolved from traditional vessel segmentation (6) and handcrafted feature extraction to *Deep Learning* (DL)-driven classification and diagnosis (7, 8). A landmark multicenter validation study by Gulshan et al. (9) published in JAMA, first demonstrated that AI systems could achieve diagnostic performance comparable to that of experienced ophthalmologists, marking the field’s entry into the translational stage. Since then, continuous algorithmic iterations – spanning *Convolutional Neural Networks* (CNNs) and *Vision Transformers* (ViTs) – alongside the integration of multimodal data such as *Optical Coherence Tomography* (OCT) and *OCT Angiography* (OCTA), have further extended AI applications from adjunctive screening to the entire care continuum (10, 11).

Despite rapid, multidimensional progress, the existing literature remains fragmented. Prior work has tended to focus on specific architectural families (e.g., *Residual Network* (ResNet) for classification, U-Net for segmentation) (12, 13), specific data modalities (14), or specific disease entities (15), without offering a systematic synthesis that integrates these disparate threads into a unified framework. In particular, the structural and temporal dynamics of the overall research ecosystem remain undercharacterized; it remains difficult to present the full evolutionary picture across time, regions, and disciplines, or to precisely identify the core knowledge base, phased hotspots, and latent research gaps. Notably, the co-occurrence patterns among AI model paradigms, data modalities, and target diseases has yet to be clearly characterized and quantitatively mapped.

Several bibliometric studies have examined the application of AI in DR from diverse perspectives (16, 17). For example, a bibliometric analysis published in a Frontiers journal traced global publications on machine learning for DR from 2011 to 2021 (18). Ghazali (19) performed a scientometric analysis of deep learning in DR image processing for 2016-2024, relying exclusively on the WoSCC. Huang et al. (20) focused specifically on AI-based DR screening using fundus images from 2014 to 2024. However, most of these studies are limited to either a single class of AI models or a single database, and none has systematically captured the co-evolutionary dynamics across three dimensions – AI model architectures, imaging data modalities, and the target disease. Notably, the coupling among these three dimensions still lacks adequate feature interpretation and quantitative mapping for visual representation.

**Figure 1:**
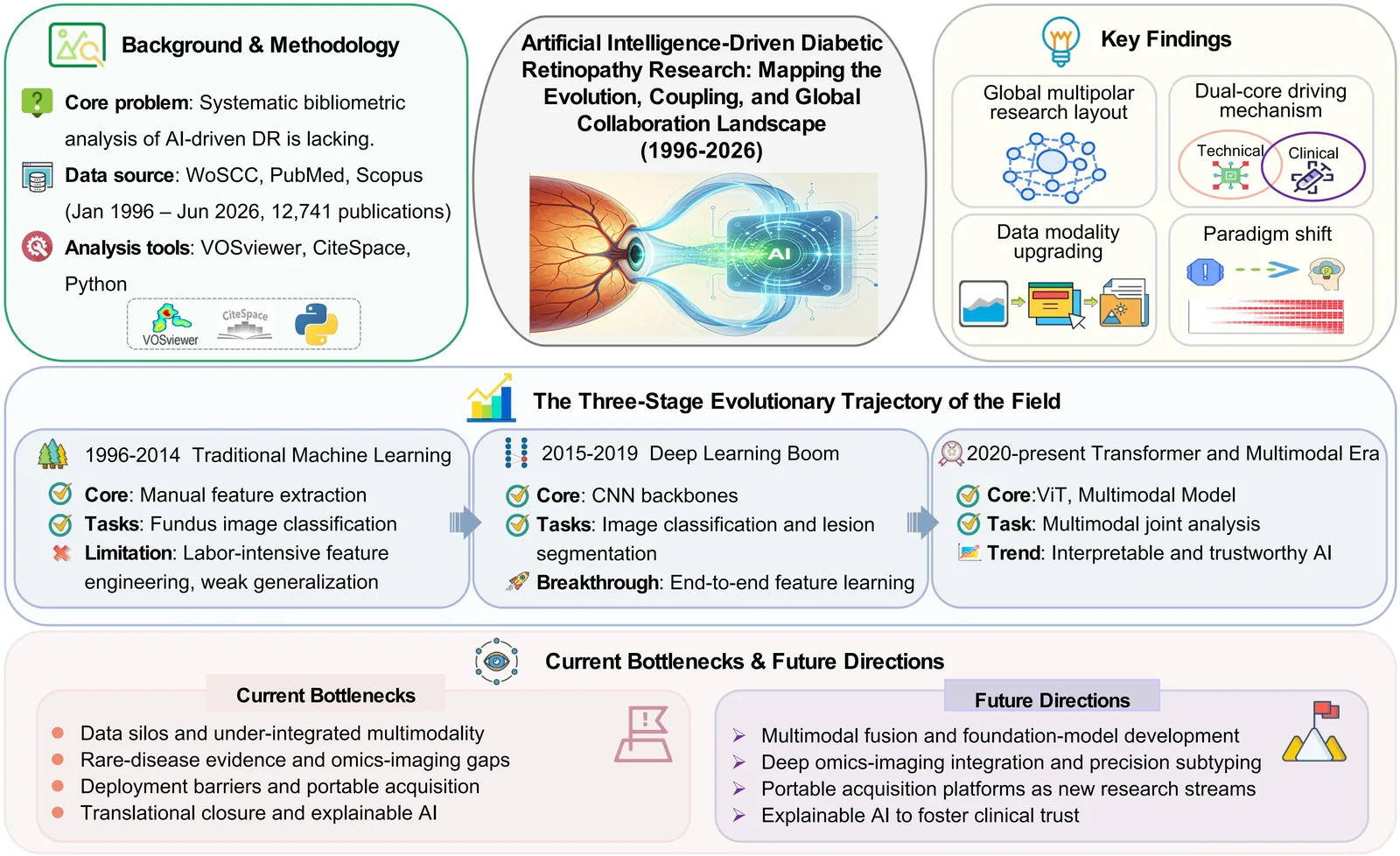
Graphical abstract of the study.

To address these gaps, the present study conducts a panoramic bibliometric and visual-analytical investigation of AI-driven DR research. By quantitatively analyzing the external attributes and content-level associations of the literature, we aim to objectively reveal the field’s developmental stages, major contributing entities, knowledge-flow pathways, and frontier trends, thereby addressing the limitations of previous narrative reviews. Drawing on 12,741 screened publications spanning from 1996 to June 2026, and integrating VOSviewer, CiteSpace, and a Python-based text-mining pipeline, this study aims to: (1) map global collaborative networks at the country, institution, and author levels; (2) identify knowledge pillars and evolutionary trajectories through co-citation analysis and keyword burst detection; (3) track the historical transitions of AI models, dominant data modalities, and core disease entities; and (4) reveal the co-occurrence patterns among these dimensions that underpin the structural backbone of the field. To our knowledge, this work provides a comprehensive roadmap for the discipline, offering researchers, clinicians, and policymakers a holistic reference for understanding the past, present, and future frontiers of AI in diabetic retinopathy. The graphical abstract (**Figure 1**) summarizes the study design.

## 2 Materials and methods

### 2.1 Literature search and screening strategy

This study sourced literature from three internationally recognized academic databases: the *Web of Science Core Collection* (WoSCC), PubMed, and Scopus. These databases facilitate standardized metadata export (e.g., full records and cited references) and exhibit high compatibility with bibliometric tools such as VOSviewer and CiteSpace, thereby providing a robust structured foundation for subsequent co-occurrence analysis, collaboration network mapping, and burst detection. The literature search was conducted on July 1, 2026. The search period spanned from January 1, 1996, to June 30, 2026, encompassing three decades to capture the complete evolutionary trajectory of the field from inception to maturation. To accommodate the distinct indexing structures across databases, this study adopted the most comprehensive search fields available in each: TS – encompassing title, abstract, and keywords – in the WoSCC, and TITLE-ABS-KEY (title, abstract, and keywords) in Scopus. For PubMed, to ensure high thematic relevance in bibliometric analysis, the search was confined to the title field, thereby focusing on literature where AI or DR represents the core research topic and improving precision. The broad searches in WoSCC and Scopus, combined with subsequent cross-database deduplication, effectively compensated for potential under-retrieval inherent in PubMed’s single-field approach. Two authors independently performed the initial screening; disagreements were resolved through discussion, with the third and fourth authors serving as arbitrators when necessary. The search strategy integrated subject headings and free-text terms. After multiple rounds of pilot testing, the final search queries are as follows:

- WoSCC: (TS=(Deep Learning) OR TS=(Machine Learning) OR TS=(Neural Network) OR TS=(Artificial Intelligence) OR TS=(Algorithm)) AND (TS=(Diabetic Retinopathy))
- PubMed: ((Deep Learning[Title]) OR (Machine Learning[Title]) OR (Neural Network[Title]) OR (Artificial Intelligence[Title]) OR (Algorithm[Title])) AND (Diabetic Retinopathy[Title])
- Scopus: (TITLE-ABS-KEY(Deep Learning) OR TITLE-ABS-KEY(Machine Learning) OR TITLE-ABS-KEY(Neural Network*) OR TITLE-ABS-KEY(Artificial Intelligence) OR TITLE-ABS-KEY(Algorithm*)) AND TITLE-ABS-KEY(Diabetic Retinopathy)

The search terms were deliberately chosen to cover the full 30-year timeframe. The core terms (“Artificial Intelligence”, “Machine Learning”, “Deep Learning”, “Neural Network”) were chosen because they represent the most foundational and consistently used umbrella terms throughout the entire study period. These terms collectively encompass the vast majority of AI methodologies applied in medical imaging and ophthalmic research. Furthermore, “Algorithm” was included as a broad catch-all term to capture early-phase AI research that predates the widespread adoption of the “Deep Learning” label, as well as studies whose primary focus is algorithmic development. Notably, publications on emerging paradigms – such as foundation models, vision-language models, and self-supervised learning – typically include at least one of our core search terms in their titles, abstracts, or keywords to signal their methodological grounding. Similar term combinations have been widely adopted in recent bibliometric studies across medical AI domains (21–23).

The initial search retrieved 17,280 records. To ensure thematic relevance and data integrity, a rigorous multi-stage screening protocol was implemented:

- Initial Screening: Records were sorted by relevance scores in descending order. Titles and abstracts were screened to exclude irrelevant studies, particularly those outside the disciplinary scope or misaligned with the research focus.
- Document Type Restriction: The analysis was restricted to original research articles, conference papers, and review articles. Non-substantive items – including editorials, letters, news items, and corrigenda – were excluded to maintain academic rigor.
- Language and Completeness Filtering: Only English-language publications were retained. Records lacking essential bibliographic fields were discarded.
- Deduplication: To avoid bias from duplicate records, this study adopted a stratified, precise matching strategy for deduplicating the records: (1) For records with a DOI, we extracted the DOI field, removed common URL prefixes (e.g., https://doi.org/, http://dx.doi.org/), converted the remainder to lowercase, and stripped leading and trailing whitespace to obtain a standardized DOI. Records with identical standardized DOIs were treated as duplicates, and only the first occurrence was retained. (2) For records lacking a DOI, this study adopted a hierarchical matching strategy. A primary composite key was constructed from three standardized fields: the title was normalized by converting to lowercase, stripping leading and trailing spaces, collapsing multiple spaces into one, and removing punctuation; the publication year was cleansed of surrounding whitespace; and the first author’s surname – extracted as the substring preceding the first comma – was reduced to lowercase. Records yielding identical primary keys were flagged as candidate duplicates. To mitigate false positives arising from metadata inconsistencies, a secondary verification step compared normalized journal names within each candidate group. Automatic deduplication was performed only when journal names matched exactly, retaining the earliest recorded entry. Records with non-matching journal names were instead preserved and flagged for manual inspection to ensure data integrity. (3) For the minority of records with incomplete metadata – such as those lacking a DOI, a valid title, or a valid publication year – no unique matching key could be generated using the aforementioned procedures. To preserve data integrity, these records were retained and excluded from the deduplication process. This conservative strategy prioritizes preventing false positives (i.e., inadvertently removing non-duplicate records) over maximizing deduplication precision.

**Figure 2:**
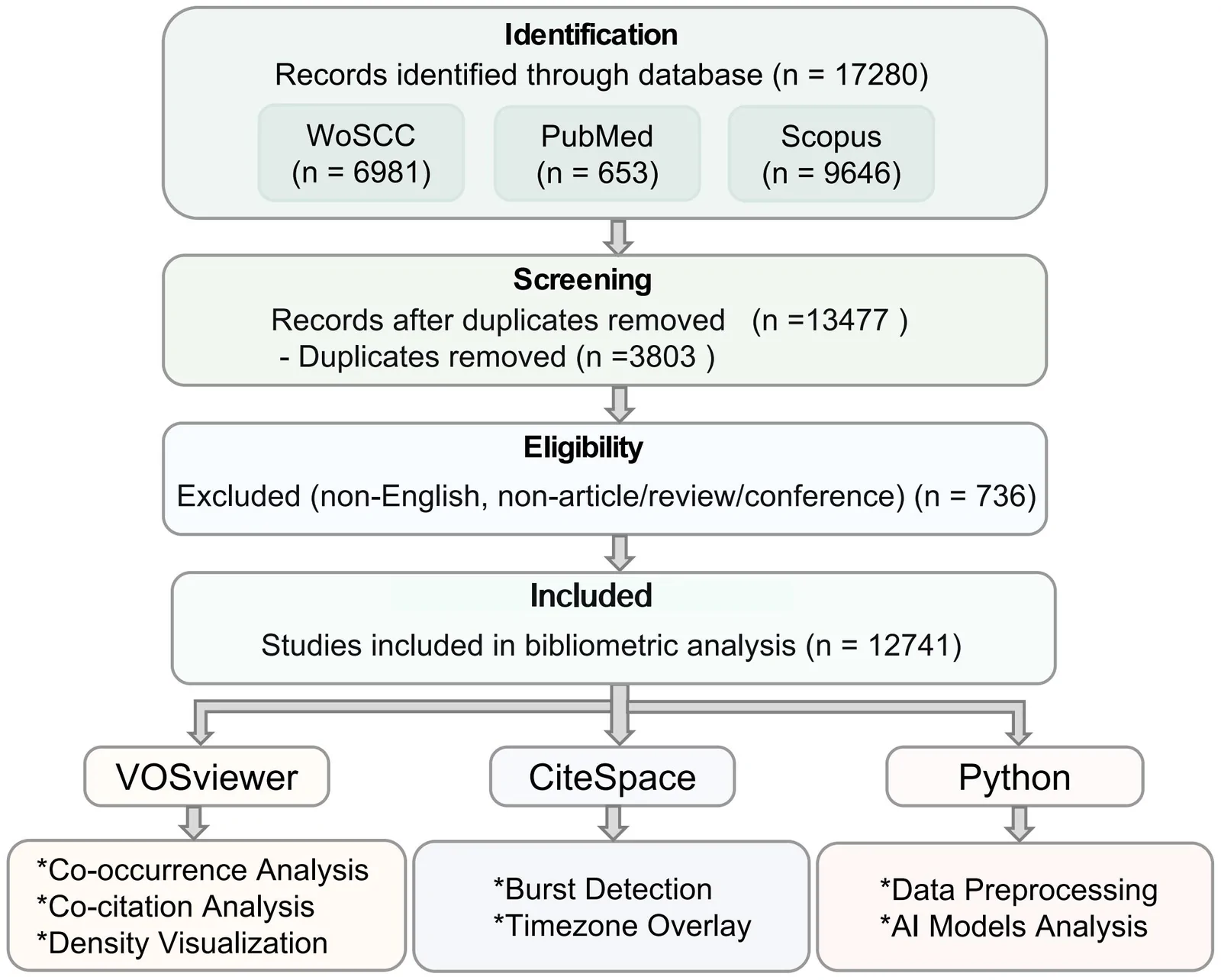
Flow diagram of literature screening.

Following this protocol, 12,741 unique records were retained. All data were exported in the WoSCC plain-text format to ensure consistency in field labeling and seamless integration with bibliometric software. The temporal distribution, document-type composition, and deduplication statistics of the final corpus are illustrated in **Figure 2**. This curated dataset constitutes the empirical basis for the subsequent bibliometric and visualization analyses.

### 2.2 Software for visualization analysis

This study employed VOSviewer (1.6.20), CiteSpace (6.4.R1), and Python 3.9 as the primary tools for bibliometric analysis and scientific knowledge mapping. To ensure the reproducibility of the analytical results and the validity of the conclusions, all software tools were configured with explicit and traceable parameter settings. The specific configurations and their theoretical justifications are detailed below.

#### 2.2.1 VOSviewer parameter settings

The parameter settings cover core analytical dimensions, including authors, sources, institutions, references, and keywords.

- Author Analysis (≥ 20 publications, ≥ 1 citation): A publication threshold of 20 papers is applied to focus on active researchers with sustained scholarly output and established academic track records, thereby preventing network fragmentation caused by a large cohort of low-productivity authors. The citation requirement (at least one citation) serves as a baseline impact filter, ensuring that

included authors possess fundamental academic visibility while balancing quantitative productivity with qualitative impact.

- Source Analysis (≥ 20 publications, ≥ 2 citations): As the primary vehicles for disseminating research, journals are analyzed to identify the core journal cluster in the field. The threshold of ≥ 20 publications screens for journals with steady and consistent output—typically the primary outlets to which scholars submit their work and that they frequently consult. The elevated citation threshold further ensures that these journals not only publish prolifically but also feature articles with demonstrable citation impact.
- Institution Analysis (≥ 20 publications, Top 50): Given that institutions typically produce significantly higher publication volumes than individuals, a threshold of 20 publications is employed to identify major research contributors. Furthermore, restricting the analysis to the Top 50 institutions controls network scale, ensuring the visualization highlights the most influential institutions while avoiding graphical clutter caused by an excessive number of nodes.
- Reference Analysis (Top 50 most highly cited references): Co-citation analysis is conducted to map the intellectual base of the field. Selecting the 50 most frequently cited references captures the field’s most widely recognized classic works. Capping the node count at 50 effectively balances information coverage with visual clarity, rendering the core knowledge structure readily discernible.
- Keyword Analysis (occurrence ≥ 10 times, Top 100): Keyword co-occurrence networks are utilized to identify research hotspots and thematic structures. A minimum occurrence threshold of 10 filters out sporadic or non-standard terms appearing only once or twice, thereby highlighting representative and recurrent research themes. Retaining the top 100 keywords then controls the final network size while preserving analytical granularity, enabling a clear visualization of the macro-level thematic architecture.

For network normalization, the LinLog algorithm was adopted to enhance strong links and suppress weak ones, while modularity-based clustering was applied to automatically partition communities based on modularity maximization, thereby clearly revealing the intrinsic structural hierarchy of the network.

#### 2.2.2 CiteSpace parameter settings

The configuration focused on time-series evolution and burst detection.

- Time slicing: The period was divided into annual slices from 1996 to 2026. Within each slice, the top 50 most cited or most frequent items (Top N = 50) were selected to dynamically track annual research frontiers, balance the data volume across periods, and avoid biases caused by low citation counts in early years or the dominance of recent publications. The parameter Top N = 50 is chosen to include a sufficient number of nodes to ensure robust and reliable clustering results, while also maintaining good visual readability of the map.
- Burst detection: The Kleinberg burst detection algorithm was applied with the following parameters: weight function *f*(*x*) = 2, number of states=2, sensitivity coefficient *γ* = 1.0, and minimum duration=2 years. This two-state model, combined with frequency-squared weighting, uses standard sensitivity to balance detection thresholds, while the minimum duration threshold excludes single-year random pulses. Setting the sensitivity coefficient *γ* to 1.0 achieves a reasonable balance between sensitivity and specificity, while the 2-year minimum duration threshold effectively filters out transient popularity fluctuations. This configuration robustly captures sustained hotspots and suppresses sporadic noise.

#### 2.2.3 Python 3.9

Python 3.9 was responsible for data preprocessing, tracking the evolution of AI models, and supplementary visualization tasks. Core dependency libraries included Pandas, re, SciPy, NumPy, as well as Matplotlib, Seaborn, Plotly, and PdfPages. The detailed analytical workflow based on Python will be elaborated in Section 2.3.

### 2.3 AI model name extraction and normalization method

**Figure 3:**
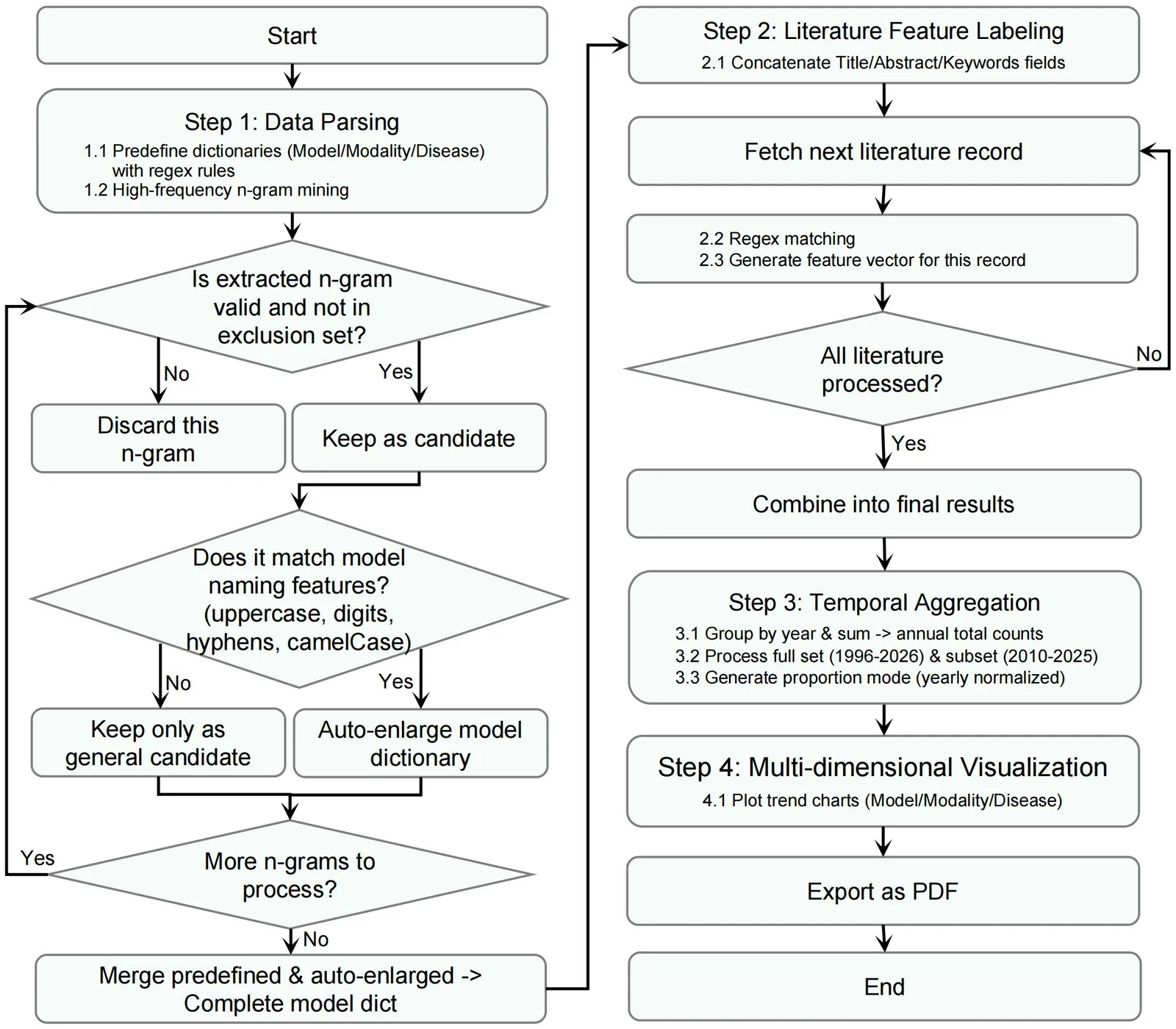
Flowchart for AI model name extraction, standardization, and trend analysis.

This study employs a hybrid approach combining dictionary-based matching and regular expression pattern recognition to extract and normalize AI model names, data modalities, and disease entities. By integrating exact matching, regular expression pattern recognition, and automated candidate mining, this approach ensures accuracy and consistency in identification across heterogeneous literature corpora. The workflow is illustrated in **Figure 3**, with key steps detailed as follows.

#### 2.3.1 Dictionary construction and keyword matching

##### 2.3.1.1 Domain-specific dictionary construction:

This study constructed a domain-specific dictionary covering AI models, data modalities, and disease entities via three sequential procedures:

- A base term list was extracted from domain review articles to form a seed dictionary.
- Each entry in each of the three dictionaries (AI models, data modalities, disease entities) was associated with a regular expression pattern to enable flexible matching of full names, abbreviations, and variants.
- The dictionaries were automatically expanded through a preprocessing phase (as detailed in Section 2.3.3), with validated candidate terms being incorporated into their respective dictionaries.
- Taking the model dictionary as an example, the regular expression design covers the following naming conventions:

- Uppercase abbreviation patterns (e.g., \bSVM\b, \bCNN(?:s)?\b) for matching standard abbreviated forms.
- Full-name patterns (e.g., Support Vector Machine, Convolutional Neural Network(?:s))? for matching complete algorithm names.
- Architecture naming patterns (e.g., ResNet(?:[-_]?\d+)?, EfficientNet(?:[-_]?B?\d+))? for matching architecture names with version numbers.
- Composite and variant patterns (e.g., U-Net\b|UNet, R-CNN\b|Faster R-CNN|Mask R-CNN) for matching hyphenated variants and composite model names.

##### 2.3.1.2 Keyword matching

For each article, the title (TI), abstract (AB), author keywords (DE), and Keywords Plus (ID) fields were concatenated into a single text string. All regular expression patterns in the dictionary were then iteratively applied to perform matching. A successful match flagged the corresponding category with a value of 1. Consequently, a single article could match multiple AI models, imaging modalities, and disease entities simultaneously.

##### 2.3.1.3 Ambiguity resolution

For ambiguous abbreviations such as “CT”, we explicitly define it in the data modality dictionary as *choroidal thickness* (matched by the pattern r’\bCTs?\b|\bchoroidal thickness\b’), thereby disambiguating the term in favor of *choroidal thickness* within ophthalmology literature, rather than *computed tomography*.

#### 2.3.2 Synonym normalization

The dictionary design inherently enables automatic normalization of synonyms, mapping all variants of the same model to a single canonical key name via regular expressions. For instance, variants – e.g., \bSVM\b, Support Vector Machine, and support vector machines – are normalized to SVM; CNN(?:s)?, Convolutional Neural Network(?:s)?, and ConvNet to CNN; ResNet(?:[-_]?\d+)? (covering ResNet-50, ResNet-101, and ResNet-152) to ResNet; and ViT(?:s)?, Vision Transformer(?:s)?, ViT-Base, and ViT-Large to ViT. The same normalization strategy is applied to data modalities and disease entities, ensuring consistency in statistical dimensions.

#### 2.3.3 Automatic dictionary expansion mechanism

To improve dictionary coverage and mitigate the risk of omitting emerging terms, an automatic expansion pipeline based on high-frequency candidate term mining was implemented.

- Candidate term extraction: A bag-of-words model was used to extract high-frequency *n*-grams with *n* ranging from 1 to 4 from the corpus. A custom stopword list – supplemented with domain-generic terms such as “patient”, “study”, “analysis”, “result”, and “method” – was applied to filter out non-informative phrases.
- Orthographic feature filtering: A heuristic function was employed to screen candidate terms based on orthographic features such as the presence of uppercase letters (e.g., ResNet), digits (e.g., VGG16), hyphens or underscores (e.g., U-Net), and CamelCase notation (e.g., DeepLab).
- Import step: For the automatically filtered candidate terms, generate the corresponding regular expression patterns and then import them into the dictionary.

The overall process for identifying models, data modalities, and disease entity names is detailed in **Algorithm 1**, which covers dictionary construction, synonym normalization, automatic dictionary expansion, and relation extraction. This pseudocode maps directly to the full Python source code and can serve as an independent implementation reference for reproducing the method.

Algorithm 1

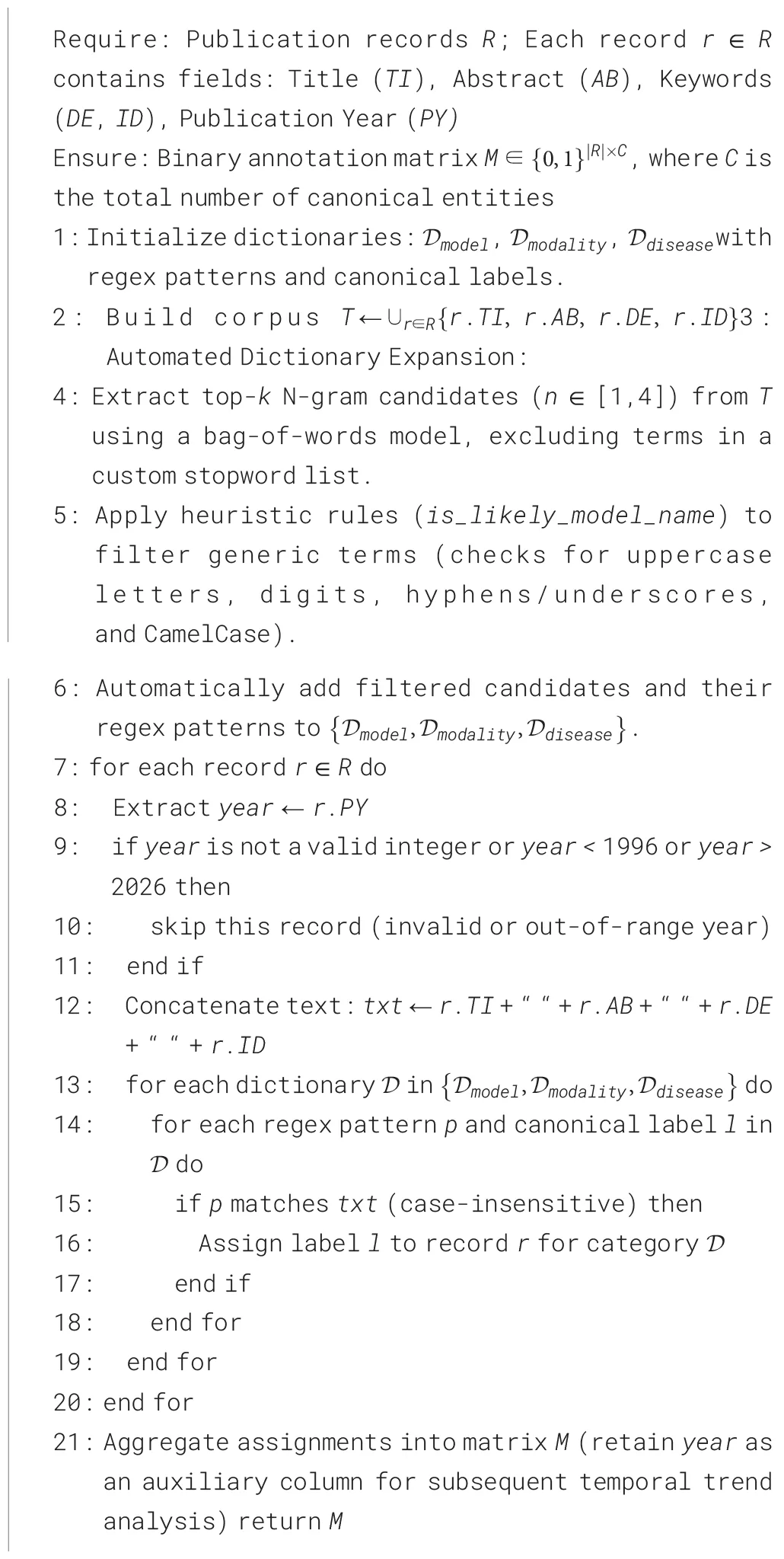

#### 2.3.4 Validation

To assess extraction accuracy, 200 articles were randomly sampled. Two authors independently annotated mentions of AI models, imaging modalities, and target diseases in the titles, abstracts, and keywords. Inter-rater reliability was evaluated using Cohen’s *κ*, which indicated strong agreement across the three categories (*κ* = 0.83, 0.85, and 0.81, respectively). Discrepancies were resolved through discussion involving the third and fourth authors to establish a consensus ground truth. The automated extraction performance was benchmarked against these labels, achieving precision values of 0.89 (models), 0.92 (modalities), and 0.87 (diseases). Primary error sources included: (a) the limited coverage of the extraction scope; and (b) ambiguous abbreviations arising from non-AI contexts. Such ambiguities (e.g., “CT”) were resolved by constraining dictionary entries to ophthalmology-specific senses during entity recognition.

## 3 Results

### 3.1 Contribution of countries and institutions

**Figure 4:**
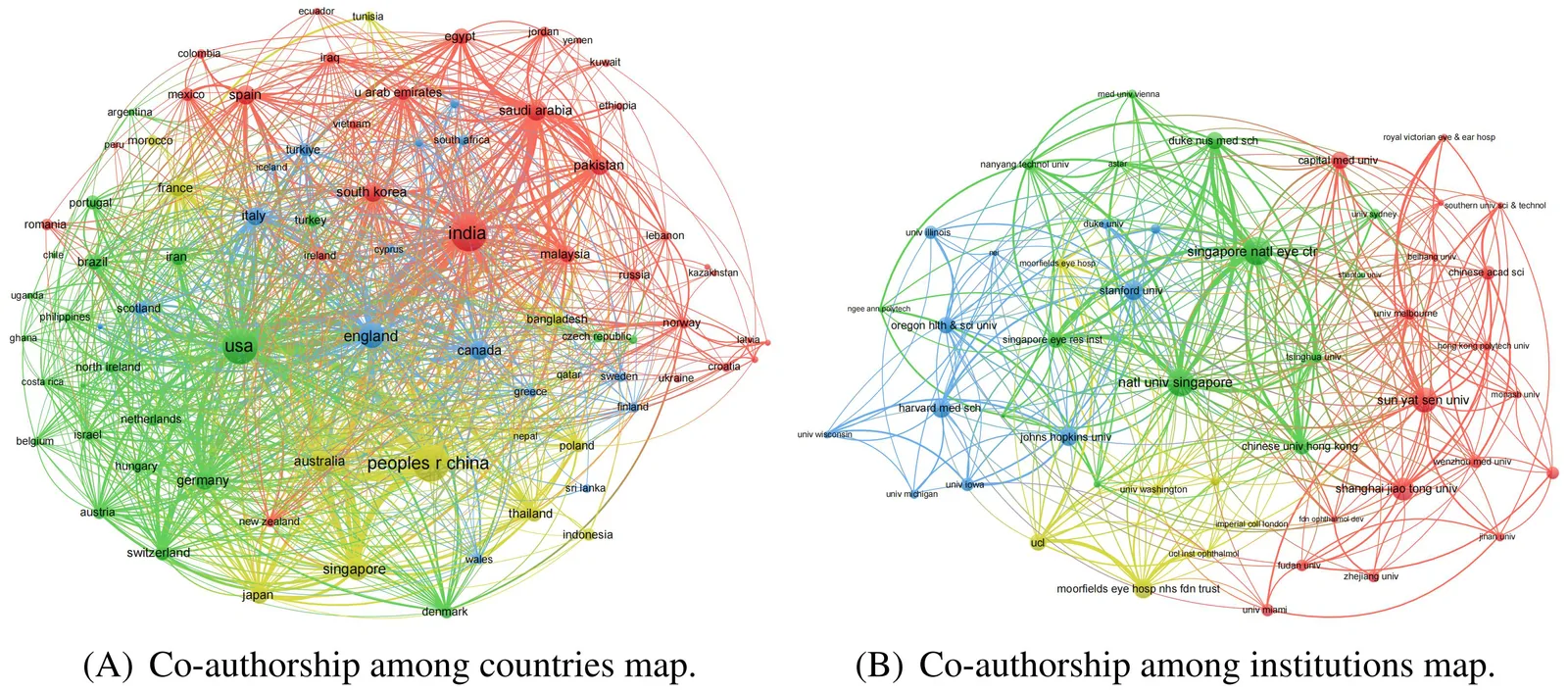
Co-authorship among countries and institutions. **(A)** Network map of co-authorship among countries. **(B)** Network map of co-authorship among institutions. (Node size reflects the number of publications of countries/institutions; line thickness indicates collaboration strength between countries/institutions; and node color represents different collaborative communities).

**Figure 4A** shows that the *United States* (U.S.), China, and India are the top three countries in publication output and constitute the first tier of productive nations, functioning as core hubs in the global collaboration network. The *United Kingdom* (UK) acts as a bridge connecting Europe, the Americas, and Commonwealth countries. Singapore, Switzerland, Austria, and Australia achieve notable citation impact, reflecting concentrated high-impact outputs and active international engagement. Saudi Arabia, the United Arab Emirates, Egypt, Pakistan, and Malaysia also appear on the list. Although these Middle Eastern and Southeast Asian countries have relatively modest publication volumes, they each exhibit measurable international collaboration linkage strength – reflecting both the global.

**Figure 4B** illustrates the institutional collaboration network. The National University of Singapore, the Singapore National Eye Centre, and Duke-NUS Medical School form a tightly integrated core cluster, indicating that Singapore leverages its concentrated research strength to serve as a major international collaboration hub in AI-driven DR research. Among Western institutions, Stanford University, Harvard Medical School, and Johns Hopkins University exhibit exceptional citation impact, functioning as independent high-impact centers. In the UK, Moorfields Eye Hospital and multiple nodes within the *University College London* (UCL) system – including UCL, the UCL Institute of Ophthalmology, and Moorfields NHS Trust – reflect robust institutional linkages and close interdisciplinary collaboration in ophthalmic clinical research. Regarding Chinese institutions, the Chinese University of Hong Kong and Sun Yat-sen University are highly active, while Shanghai Jiao Tong University, Capital Medical University, and 302 Tsinghua University also demonstrate significant engagement in international collaborations.

### 3.2 Analysis of authors

**Figure 5:**
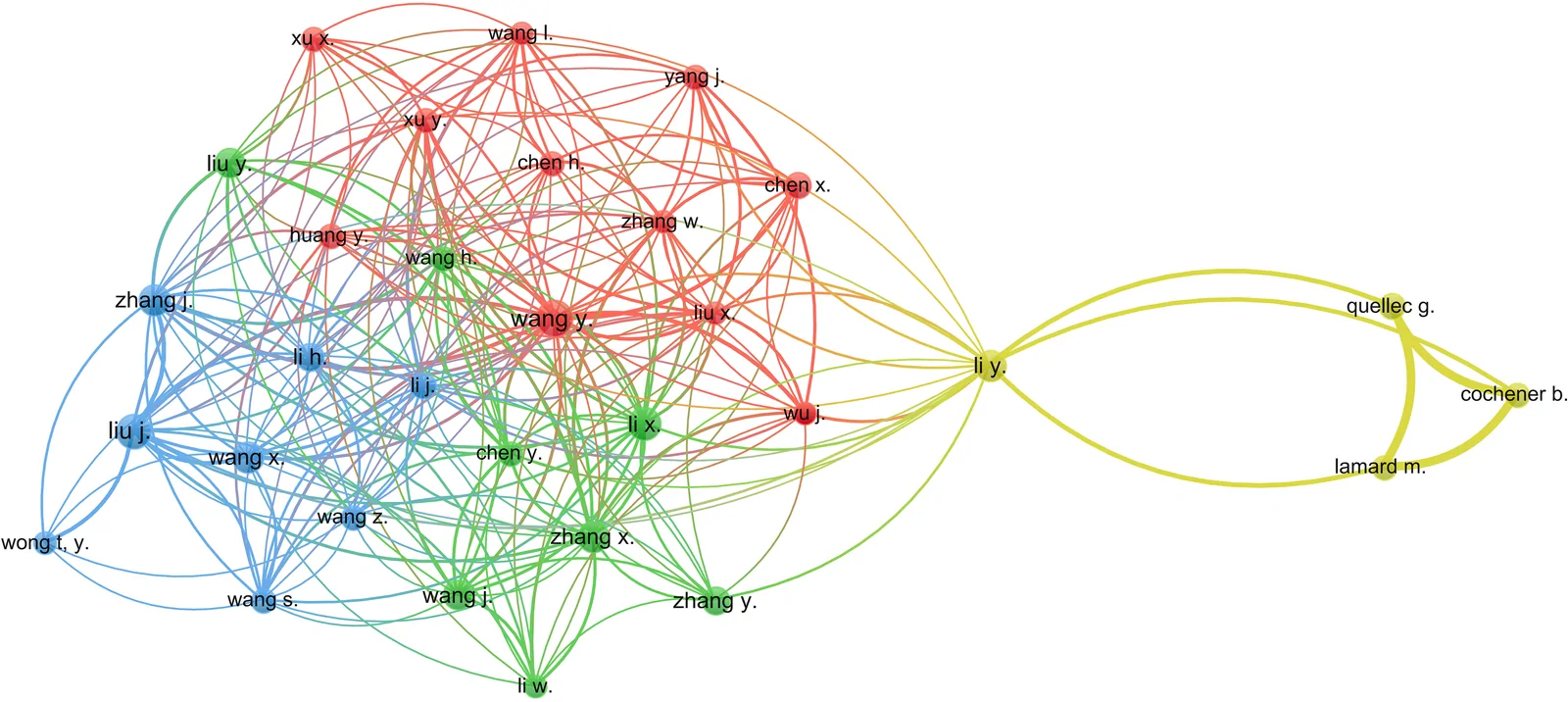
Co-authorship among authors map (node size represents the number of documents published by each author; link thickness indicates the frequency of co-authorship; and node color denotes the collaborative cluster).

**Figure 5** shows several distinct dense clusters in the author collaboration network, indicating that research activity in this field is strongly clustered along geographic and institutional lines. The Asia-Pacific core clusters (Clusters 1 and 3, in red and blue) contain the largest nodes and densest connections, representing the most active hubs of global AI-driven DR research, with prolific authors such as Wang Y. and Liu J. at the center of each cluster. A distinct European algorithm and device cluster (Cluster 4, in yellow), centered on Quellec G., Lamard M., and Cochener B., demonstrates remarkably strong internal cohesion, reflecting Europe’s extensive expertise in foundational medical imaging algorithms and device development. Another Asia-Pacific sub-cluster (Cluster 2, in green), comprising scholars such as Li X., Zhang X., and Wang J., is densely connected and forms an active sub-network focused on large-scale DR screening and epidemiological studies. AI-driven DR research exhibits a multipolar distribution, forming several distinctive research clusters. European scholars (e.g., Quellec G. et al.) occupy a bridging position across clusters, disseminating Europe’s rigorous algorithm-evaluation standards to regions with richer clinical datasets while simultaneously gaining reciprocal access to these data, thereby facilitating cross-regional knowledge flow. The current landscape is still shaped by bilateral interactions between the Asia-Pacific and European regions. However, the exclusion of high-burden regions such as Africa and South America from the core network is a gap that demands attention; future international cooperation should be more inclusive and oriented toward global health equity. Notably, among the most prolific authors, publication volume does not consistently translate into per-paper citation impact, suggesting that the field’s next phase may benefit from prioritizing generalizability over output scale.

**Table 1** lists the top 15 authors ranked by the number of documents. Productivity and impact are both evident among these top authors, though the two metrics do not always align. For instance, Liu J. (47 papers, 1,539 citations, avg 32.74) and Chen X. (27, 1,275, 47.22) show high per-paper impact, while Wang Y. (54, 844, avg 15.63) and Zhang X. (40, 601, avg 15.03) produce more papers but receive lower average citations. Li Y. (39, 934, avg 23.95) and Li H. (31, 1,011, avg 32.61) also exhibit relatively high influence. In contrast, authors such as Li J. (26, 208, avg 8.00) and Huang Y. (25, 269, avg 10.76) have notably lower citation averages. Notably, Quellec G. (26 papers, 334 citations, avg 12.85) has a total link strength of 53, which is the second-highest in the table (only Wang Y. has 66), suggesting that he maintains strong collaborative ties despite a comparatively modest per-paper impact.

**Table 1:** Top 15 Authors ranked by documents.

Author	Documents	Citations	Total link strength	Citations per document
wang y.	54	844	66	15.63
liu j.	47	1539	49	32.74
li x.	43	676	47	15.72
zhang x.	40	601	42	15.03
zhang j.	40	835	40	20.88
wang x.	40	836	29	20.90
li y.	39	934	45	23.95
liu y.	37	1017	23	27.49
wang j.	34	697	26	20.50
zhang y.	32	454	16	14.19
li h.	31	1011	27	32.61
chen x.	27	1275	24	47.22
quellec g.	26	334	53	12.85
li j.	26	208	35	8.00
huang y.	25	269	25	10.76

### 3.3 Analysis of references and sources

**Figure 6:**
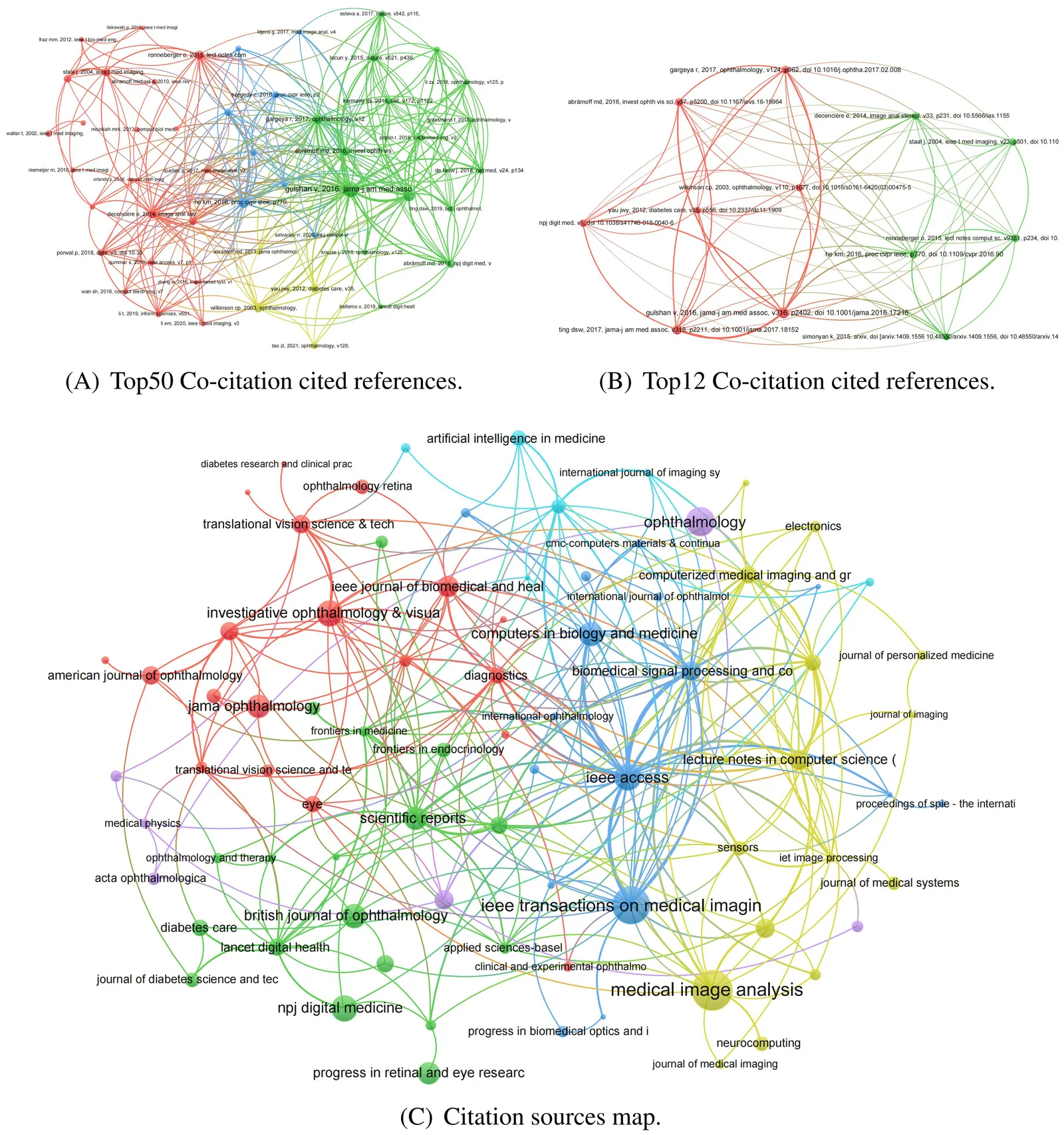
Co-citation cited references and sources. **(A)** Co-citation network of the top 50 cited references. **(B)** Co-citation network of the top 12 cited references. **(C)** Co-citation map of citation sources. (Node size reflects the citation count of documents or the publication output of journals; line thickness indicates the strength of association between documents or journals; and different node colors correspond to different collaborative thematic groups).

**Figure 6A** presents the core literature clusters, and **Figure 6B** lists the top 12 cited publications, together revealing the hierarchical structure and knowledge evolution of the AI-driven DR co-citation network. In terms of knowledge-base composition, the core literature falls into four strands: (1) Clinical AI diagnostic systems (e.g., 9, 24–28) – large-scale validation studies in clinical journals that act as primary knowledge hubs; (2) Computer-vision foundations (e.g., 29 on ResNet; 30 on U-Net; 31 on *Visual Geometry Group* (VGG); 32 on Inception); (3) Fundus-image, segmentation benchmarks, and explainability methods (e.g., 33–36, which shifts the discourse from “whether AI detects” to “why it detects” via explainable localization; (37), Indian DR dataset; and (38), reflecting recent advances in deep ensemble learning for DR screening), which supply data benchmarks, segmentation annotations, and evaluation protocols; and (4) Clinical context, disease burden, and core grading standards (e.g., 39 on ETDRS, and 40, which frames the epidemiological motivation). Within this echelon, Gulshan et al. (9) stands out as the seminal piece and principal dissemination hub, followed by Ting, Gargeya, and Abramoff’s clinical-validation cluster. At the data/standard layer, Staal, Hoover, Decencière, and Porwal provide segmentation annotations and benchmark corpora, whereas Wilkinson anchors the ETDRS grading schema. Notably, newer entries signal diversification:

Kermany et al (41), Cell) pioneers transfer learning for OCT/DR classification; De Fauw et al. (42), Nat Med) is DeepMind’s landmark integrating segmentation and diagnosis.

Based on the citation source analysis in **Figure 6C**, the AI-driven DR research field exhibits a distinct dual-core structure characterized by “engineering/algorithmic cohesion” and “clinical validation authority”. The engineering core – represented by IEEE Transactions on Medical Imaging, Medical Image Analysis, and LNCS (including MICCAI) – carries fundamental algorithmic innovations in DL-based segmentation and feature extraction, forming dense hubs within the knowledge network. The clinical translation core – represented by The Lancet Digital Health and Ophthalmology – establishes the evidence chain from “algorithmically feasible” to “clinically usable” through landmark large-scale prospective validation studies, with IOVS further reinforcing foundational medical support for disease definition and fundus grading. Notably, high-frequency co-citation links between method-oriented and clinically oriented clusters – channeled also through broad-scope venues such as Scientific Reports – map the efficient flow of knowledge along the path: disease burden assessment → algorithm proposal → engineering adaptation → clinical validation. This highly coupled interdisciplinary pattern confirms the deep coupling between technological development and clinical needs, and signals that future breakthroughs will rely increasingly on synergistic innovation across computer science and clinical medicine rather than on isolated advances within a single discipline.

**Table 2** reveals a trend toward interdisciplinary convergence in AI-driven DR research. Among engineering venues with high publication volumes, IEEE Access (175 papers) and Lecture Notes in Computer Science (191 papers) contributed substantial output but recorded relatively low citation counts per paper (32.87 and 14.18, respectively). In contrast, clinical and biomedical journals such as Ophthalmology (130.71), The Lancet Digital Health (104.95), and npj Digital Medicine (109.39) yielded substantially higher citations per paper, despite their more modest publication volumes. Notably, specialized engineering journals including Medical Image Analysis (291.00) and IEEE Transactions on Medical Imaging (176.61) also demonstrated remarkably high citation counts per paper. Furthermore, broad-scope open-access journals such as Scientific Reports (194 papers; 4,031 total citations) reflect the field’s extensive multidisciplinary reach.

**Table 2:** Top 20 Sources by citation.

Source	Documents	Citations	Citations per document
medical image analysis	51	14841	291.00
ieee transactions on medical imaging	69	12186	176.61
ophthalmology	49	6405	130.71
ieee access	175	5752	32.87
npj digital medicine	49	5360	109.39
investigative ophthalmology & visual science	230	5118	22.25
computers in biology and medicine	95	4428	46.61
british journal of ophthalmology	78	4156	53.28
scientific reports	194	4031	20.78
jama ophthalmology	71	3768	53.07
progress in retinal and eye research	25	3465	138.60
ieee journal of biomedical and health informatics	65	3356	51.63
lecture notes in computer science	191	2709	14.18
computer methods and programs in biomedicine	52	2668	51.31
biomedical signal processing and control	185	2462	13.31
plos one	103	2459	23.87
translational vision science & technology	99	2288	23.11
biomedical optics express	63	2167	34.40
american journal of ophthalmology	47	2120	45.11
lancet digital health	20	2099	104.95

### 3.4 Analysis of keywords

**Figure 7:**
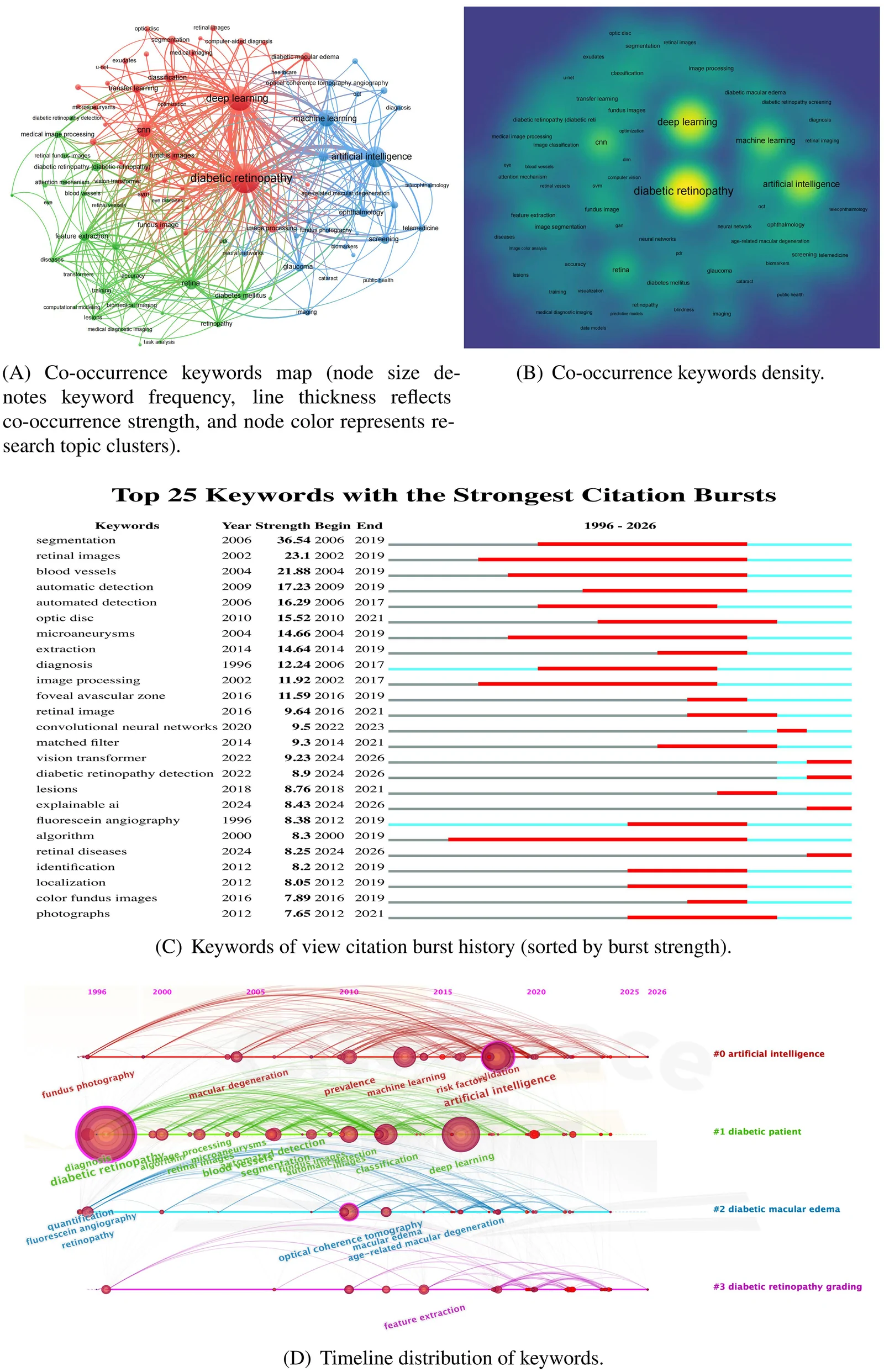
Result of co-occurrence keywords. **(A)** Keyword co-occurrence network map, displaying the correlations and clustering among high-frequency keywords. **(B)** Keyword density map, illustrating the distribution intensity of research hotspots. **(C)** Top keywords with the strongest citation bursts, highlighting emerging research trends over time. **(D)** Timeline view of keywords, showing the evolution and duration of research topics across different years.

Based on keyword co-occurrence analysis, **Figures 7A, B** present the keyword network of this field, which exhibits a radial structure centered on “diabetic retinopathy”, with “deep learning” and “artificial intelligence” as the dominant technical terms. Within this network, “accuracy” and “classification” show both high frequency and strong link strength, indicating that model verification and classification tasks constitute the most active research foci. In contrast, “segmentation” and “CNN” underpin the technical pipeline in terms of image partitioning and model architecture, respectively. Notably, the co-occurrence of “optical coherence tomography” and “fundus images” signals that multimodal image fusion is an emerging trend. Furthermore, the prominence of “diabetes mellitus” confirms that disease burden assessment serves as a consistent entry point, while the clustering of comorbidity-related terms – such as “glaucoma”, “macular degeneration”, and “macular edema” – indicates that AI-driven DR research is expanding into broader differential diagnosis and generalizability across retinal diseases.

**Figure 7C** presents the keyword burst analysis, which outlines the technological evolution of AI in diabetic retinopathy research – from traditional image processing to deep learning dominance, and onward to next-generation AI architectures. Early bursts centered on high-intensity basic image-processing tasks such as “segmentation” and “blood vessels”, laying the foundation for algorithmic lesion detection. Subsequently, the research focus shifted toward clinical applications of “automatic detection” and “diagnosis”, marking early attempts to implement assisted diagnostic systems. Most recently, building on “convolutional neural networks” and followed by the emergence of “vision transformer” and “explainable AI” starting in 2024, the research field charts the current and future core trajectories: a comprehensive shift from traditional machine learning to deep learning architectures, together with growing efforts to address the model “black-box” problem, thereby advancing AI-based diagnosis toward a more accurate, transparent, next-generation paradigm.

The keyword timezone view in **Figure 7D** delineates the evolutionary trajectory of AI-based diabetic retinopathy diagnosis, tracing its progression from conventional clinical practice to intelligent healthcare. During the early phase (1996-2005), research focused on traditional image-processing tasks (vessel segmentation, microaneurysm detection) and quantitative grading under traditional machine learning. The middle phase (2006-2014) marked a shift toward quantitative grading and traditional machine learning algorithms. After 2014, the field entered a period of deep learning expansion, with CNNs enabling automated lesion detection and feature extraction. In recent years (2020-present), research has advanced toward multimodal fusion and precision medicine, emphasizing federated learning, explainable AI, and predictive screening for cardio-cerebrovascular complications – reflecting a trend defined by the deep integration of technological iteration and clinical demand.

### 3.5 Analysis of hotspots migration

#### 3.5.1 Analysis of models

**Figure 8:**
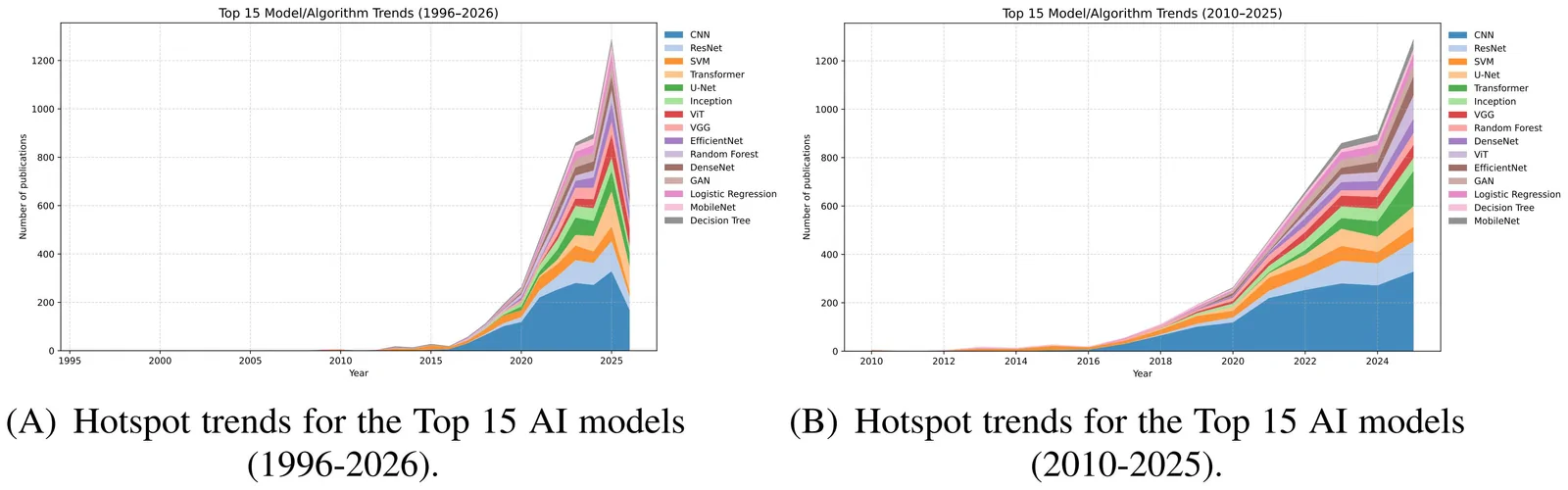
Hotspot trends for the Top 15 AI models. **(A)** Hotspot trends from 1996 to 2026. **(B)** Hotspot trends from 2010 to 2025.

**Figure 8A** illustrates the trend in applying AI models to DR over the years. For clearer observation, we also plot the trend from 2010 to 2025 in **Figure 8B**. Based on publication volume, AI-driven DR diagnosis research has undergone three distinct phases:

Traditional machine learning era (∼ 1996-2014): Traditional shallow models such as SVM and logistic regression predominated. Due to constraints in computational power and data scale at the time, researchers relied mainly on handcrafted features (color, texture, morphological attributes) from fundus images fed into classifiers. Although accuracy was acceptable, labor-intensive feature engineering limited generalizability to complex lesions.

Deep learning surge (∼ 2015-2019): With CNNs (e.g., AlexNet, VGG, GoogLeNet) rising to prominence through ImageNet, DR diagnosis leapt forward. From ∼2016, CNN-based publications grew rapidly. CNNs’ automatic feature extraction allowed models to learn pathology directly from raw fundus images, substantially reducing the missed diagnosis rate.

Transformers and vision foundation models rising phase (∼ 2020-present): The most striking trend is the increase in publications on Transformers and their variants (e.g., ViT, Transformer) since 2020. The proposed driving factor is the self-attention mechanism, which can theoretically capture long-range spatial dependencies in fundus images (e.g., associations between tiny hemorrhages and extensive exudates). Preliminary retrospective studies suggest that this mechanism may improve sensitivity to early-stage and focal DR lesions. Nevertheless, CNN-based models continue to garner substantial research attention due to their strong inductive bias and efficient local feature extraction capabilities.

#### 3.5.2 Analysis of disease entities

**Figure 9:**
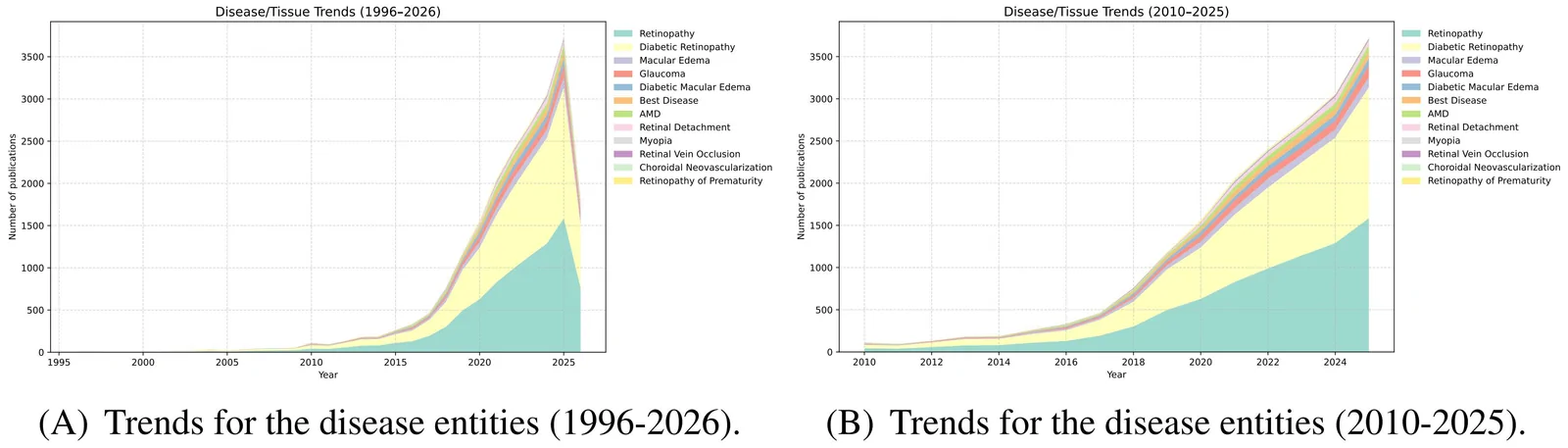
Trends for the disease entities. **(A)** Trends from 1996 to 2026. **(B)** Trends from 2010 to 2025.

**Figure 9** presents the publication trends for major diseases in ophthalmic AI research, spanning 1996-2026 (A) and 2010-2025 (B). The figure shows that AI-driven DR research accounts for the largest share of publications, while interest in other retinal diseases – such as Glaucoma, Macular Edema, *Age-related Macular Degeneration* (AMD), and Retinal Vein Occlusion – is also on the rise. Further analysis indicates that these non-DR diseases play a supplementary role in DR-related literature, which manifests in three specific scenarios: (1) Control categories for multi-class differential diagnosis – a large number of AI studies adopt multi-class classification architectures that incorporate DR alongside other similar pathologies, in order to test model specificity for DR and reduce misdiagnosis rates; (2) Comorbidity or complication contexts – in modeling the course of DR, discussions often involve complications such as Diabetic Macular Edema and hypertensive retinopathy; and (3) Generalization validation scenarios via transfer learning – given the scarcity of labeled data for rare fundus diseases (e.g., Best Disease, Retinitis Pigmentosa), some studies fine-tune models pre-trained on large-scale DR datasets to test the generalizability of AI frameworks under data-scarce conditions.

In summary, the non-DR disease entities identified in the figure should not be interpreted as indicating that the scope of this study has expanded to cover all ophthalmic diseases; rather, they serve as supplementary means to validate and enhance AI model capabilities in DR research.

#### 3.5.3 Analysis of data modality

**Figure 10:**
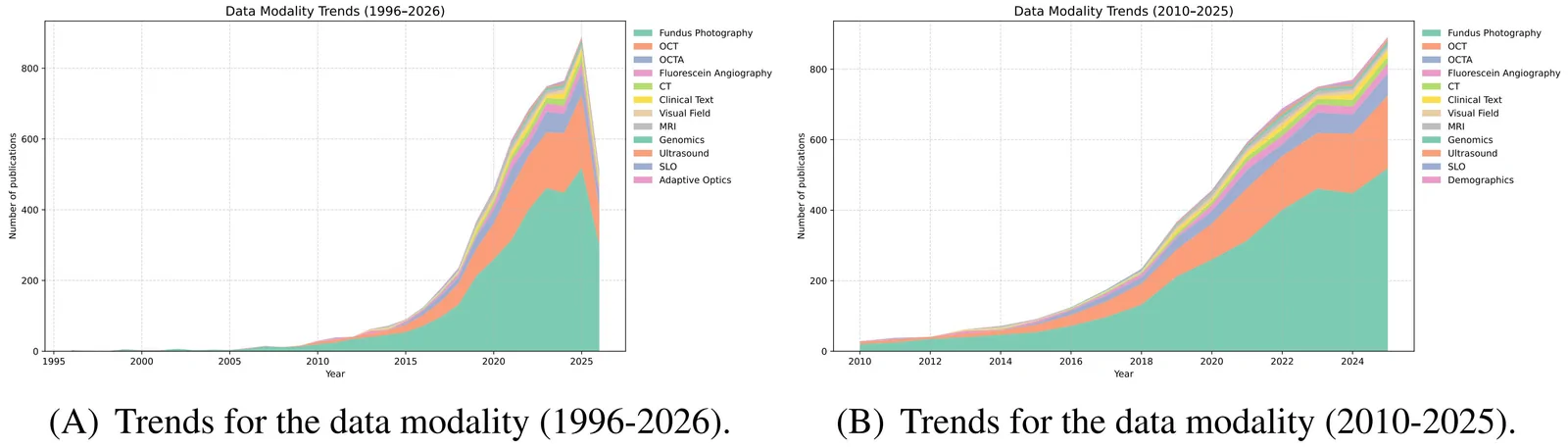
Trends for the data modality. **(A)** Trends from 1996 to 2026. **(B)** Trends from 2010 to 2025.

sources. Looking at the overall trends, all modalities show an upward trajectory. This growth pattern reflects the broad potential of multimodal data fusion in ophthalmic AI research: by integrating multidimensional data – including structural imaging, functional assessment, clinical text, and molecular features – researchers aim to build more comprehensive and robust intelligent diagnostic systems.

**Figure 10** illustrates the publication trends of various data modalities in ophthalmic AI research during 1996-2026 (A) and 2010-2025 (B). The vertical axis represents the total annual number of publications, with each stacked layer corresponding to an individual modality, and the overall stacked area reflects the cumulative output of multimodal ophthalmic data research. As shown in the legend, fundus photography accounts for the largest volume of publications, constituting the bottom layer of the chart. The OCT technology system also contributes substantially, encompassing structural imaging, quantitative parameters such as *Choroidal Thickness* (CT), and the derived angiographic technique OCTA. In addition, *Fluorescein Angiography* (FA) occupies a significant share, with *Scanning Laser Ophthalmoscopy* (SLO) and demographic data extending upward from it. Other data types, including clinical text, visual field testing, and genomic data, all exhibit steady growth. The incorporation of genomics has advanced research into hereditary eye diseases, disease susceptibility genes, and molecular pathogenic mechanisms. Meanwhile, visual field testing and *Electroretinography* (ERG) are widely applied in prognostic assessment and visual function evaluation, whereas other modalities such as clinical text serve primarily as supplementary data.

#### 3.5.4 Aligning AI models, Data, and Disease

In constructing the relationship graph among the three categories – AI models, data modalities, and diseases – each term is treated as an independent unit of analysis within its respective category. Specifically, when a single article mentions multiple models and multiple modalities, these terms are counted separately within their own categories, to fully capture the association patterns reported in the literature.

**Figure 11:**
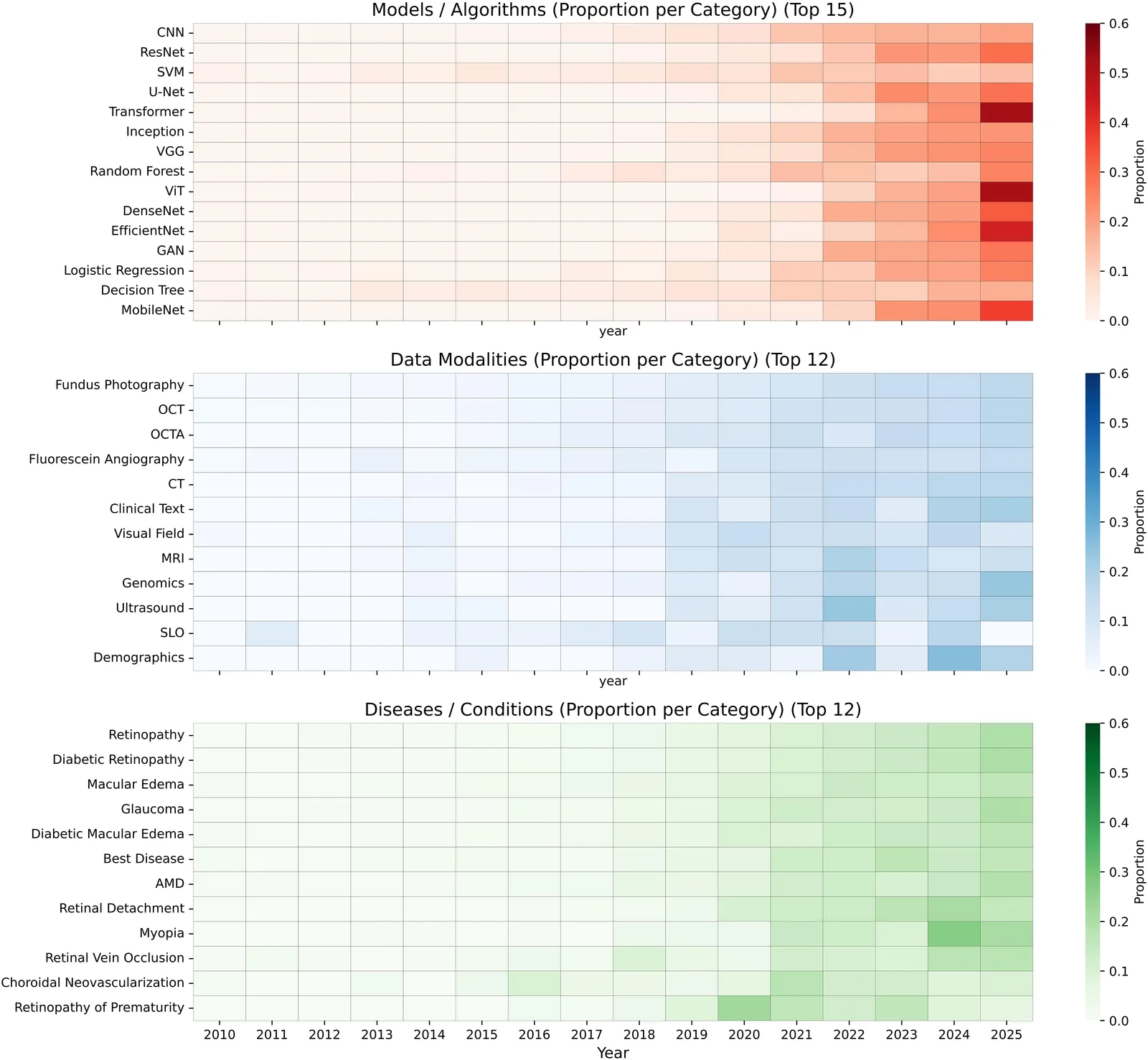
Heatmap of AI models, data modalities, and disease applicability.

**Figure 12:**
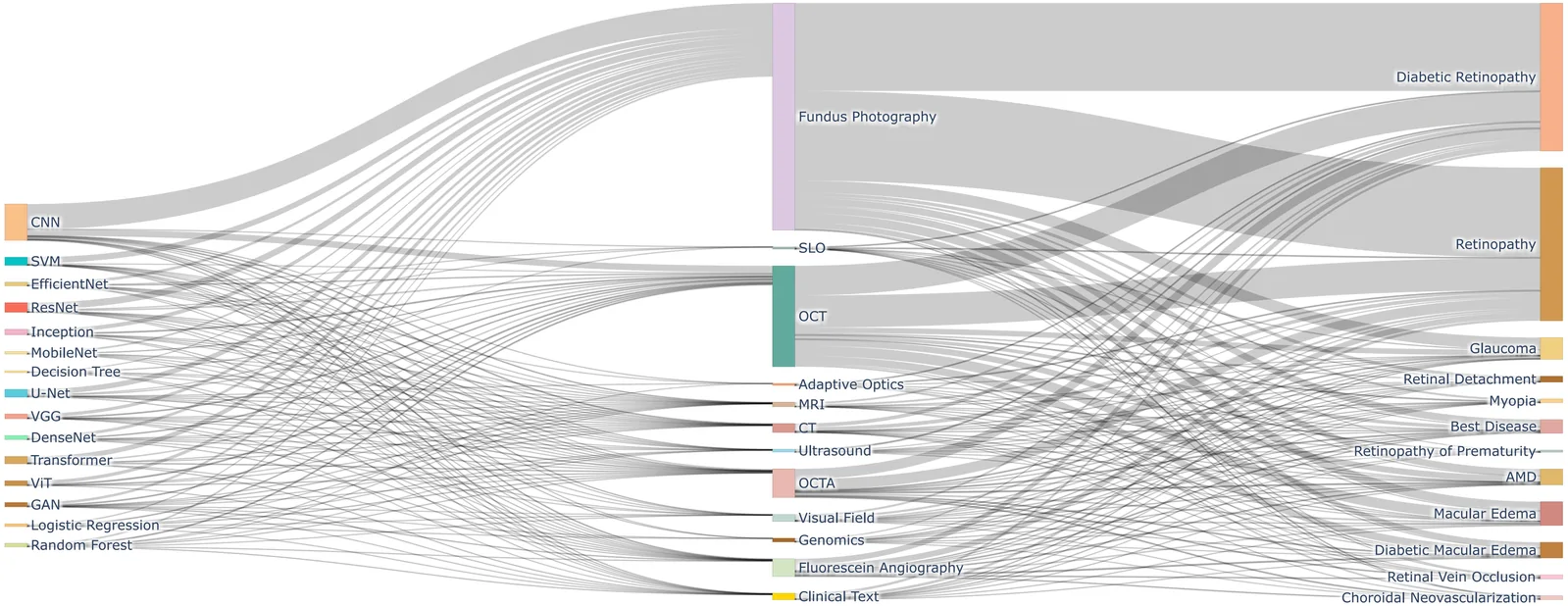
Sankey diagram: AI models → data modalities → disease (1996-2026).

We constructed three annual proportional heatmaps (**Figure 11**, Top 15 algorithmic models, Top 12 data modalities, Top 12 fundus disease types) alongside one Sankey diagram (**Figure 12**). Together, these map out the three-layer research chain “AI models → data modality → fundus/retinal disease”, enabling a quantitative temporal analysis of topic shares at each stage (2010-2025) and a dynamic visualization of element flows and association priorities across the full cycle (1996-2026). In the heatmaps, darker blocks denote a higher proportional representation for that entry in a given year; in **Figure 12**, line width corresponds to research volume. The two visualizations corroborate each other, yielding the following core findings.

AI algorithmic paradigms have undergone staged iteration. In the early period, traditional machine learning (logistic regression, SVM, random forest, decision trees) dominated, relying on handcrafted imaging features for classification – simple, interpretable, but limited in automated feature extraction. CNN-based models then became the core: ResNet and VGG served as the most widely used backbones; U-Net saw extensive uptake in image segmentation owing to its fit for fundus lesion tasks; EfficientNet and MobileNet, emphasizing lightweight design and mobile deployment, gained prominence later. The CNN family, with end-to-end feature learning, displaced manual feature engineering and established the foundational algorithmic framework for fundus AI. In recent years, ViTs and Transformer-based architectures have risen noticeably, signaling a shift from local convolutional extraction to global attention-based modeling.

Data modalities concentrate heavily in imaging, with four core sources forming the input foundation. The heatmap series shows that fundus photography, OCT, OCTA, and FA maintain consistently high shares, representing the most frequent inputs for AI modeling. In **Figure 12**, the main inflows – fundus photography, OCT, FA – are the thickest, channeling the bulk of downstream model inputs. By contrast, genomics, clinical text, visual field, ERG, and ultrasound account for smaller shares and serve mainly as supplementary data.

On the disease side, AI-driven DR research has evolved progressively along the three core dimensions: staging granularity, complication modeling, and cross-disease differentiation. In the disease heatmap, “retinopathy (broad)” and DR dominate the disease heatmap with the darkest saturation, feeding into the widest terminal flows in **Figure 12**. This confirms that the objective of AI integration remains the DR staging (e.g., distinguishing mild Non-Proliferative DR from pre-proliferative stages) and the quantitative modeling of complications. Furthermore, the inclusion of conditions such as Retinal Vein Occlusion and AMD reflects the intensive focus on cross-disease differentiation. These entities, along with Glaucoma and retinal venous occlusions, serve as critical contrast groups; their presence as narrower but distinct flows demonstrates their utility in enhancing model specificity and reducing misdiagnosis against visually similar pathologies. Rare and hereditary diseases (e.g., retinopathy of prematurity, Best Disease) and structural anomalies like retinal detachment, while representing smaller research volumes, function as essential distractor categories or robustness test cases, ensuring the generalizability of the AI frameworks originally validated in the DR domain.

Cross-layer associations show clear canonical pairings, with mainstream paradigms stabilized. **Figure 12** reveals a tendency toward recurrent pairings: the combination of CNN backbones (ResNet or U-Net) with FA or OCT predominates in studies of DR, Macular Edema, and hereditary retinal degenerations; the pairing of ViT with fundus photography or OCT is primarily applied to DR and retinal diseases in general; and Transformers combined with clinical text data represent another common pairing. Meanwhile, traditional machine learning also exhibits stable application patterns in image-based tasks, benefiting both from its role as an output-layer classifier in deep learning pipelines and from its integration with clinical text or visual field data for small-sample predictive modeling. Overall, cross-element pairings frequently co-occur, indicating that several canonical configurations have emerged in this field. Notably, the annual proportions of all elements in the chain rise in parallel, reflecting the joint expansion of algorithmic choices, data usage, and disease targets.

## 4 Discussion

### 4.1 Globally collaborative countries and institutions

The observed patterns in country and institutional collaborations suggest that publication volume alone is an insufficient metric of academic impact. While developed Western nations maintain a competitive edge in methodological innovation and groundbreaking studies, China and India leverage their demographically large populations and abundant DR clinical data to support extensive AI model training (43, 44). Nevertheless, high-quality data annotation and robust cross-center validation remain critical bottlenecks for enhancing global influence (45) – a key impetus for these nations’ active pursuit of international partnerships. The intense competition in publication output among India, China, and Singapore underscores the Asia-Pacific region’s collective response to the escalating burden of diabetes (46). Singapore is particularly noteworthy; its consistent production of high-impact research demonstrates the potential of AI to achieve precise diagnostics within standardized healthcare frameworks. Concurrently, the growing involvement of Middle Eastern countries (e.g., Saudi Arabia) signals that AI-driven fundus screening is rapidly evolving into a strategic tool for addressing specific public health demands (47). Institutionally, ophthalmic AI research often manifests as tightly integrated clusters bridging clinical, academic, and industrial sectors, as exemplified by the Singaporean ecosystem. Leading U.S. institutions also foster international collaborations, thereby enriching the global research ecosystem. Chinese institutions, despite their high publication volumes, often exhibit more fragmented collaboration networks, highlighting a need for stronger joint validation efforts and data-sharing initiatives with the global research community. Moving forward, establishing a more inclusive global collaboration network is imperative. Future endeavors should prioritize bias mitigation and algorithmic equity – ensuring efficacy across diverse ethnicities and imaging devices – through enhanced transnational and cross-ethnic data sharing. Such efforts will be pivotal in driving the global standardization and clinical implementation of AI-assisted DR diagnosis and treatment.

### 4.2 Co-citation network: knowledge foundations and evolutionary directions

Based on the co-citation analysis results (**Figure 6**), the highly linked literature delineates two core pillars of AI research in DR: clinical validation and underlying algorithms. At the level of knowledge bases and algorithmic architectures, the co-citation network reveals a clear technological evolutionary path. VGG (31) and U-Net (30) constitute the algorithmic cornerstones – the former providing a powerful paradigm for image feature extraction, and the latter emerging as a widespread framework for precise fundus lesion segmentation thanks to its encoder-decoder structure. Meanwhile, the persistence of earlier works (e.g., Quellec et al. (48)Zhang et al. (49)) suggests that traditional morphological analysis and stereoscopic fundus image processing have not been supplanted but rather integrated as key components of modern hybrid models. These algorithmic publications exhibit strong knowledge coupling with the clinical validation studies of Gulshan et al. (9) and Ting et al. (24), as well as the translational outcomes of Abràmoff et al. (26), 27), underscoring the deeply intertwined nature of “advanced algorithms” and “rigorous clinical validation”. From the perspective of research frontiers, the network maps a paradigm shift from single-disease screening to comprehensive clinical management. Early studies (e.g., Staal et al. (33) and Hoover et al. (34)) focused on vessel segmentation and morphological quantification, establishing initial standards. A surge between 2015 and 2019 saw a rapid pivot toward 538 enhancing diagnostic accuracy via deep learning. Notably, Kermany et al. (41) in Cell marked the widespread acceptance of AI-driven pathology by the life sciences community. Reflecting recent citation trends, current and future hotspots extend beyond “image recognition accuracy” to encompass model interpretability, multimodal data fusion (e.g., with OCT), and practical deployability in primary care – signaling a focus on generalizability and real-world implementation.

Overall, this network maps out a distinct research chain: disease burden assessment → basic image processing → deep feature extraction → large-scale clinical validation → exploration of interpretability and generalizability. The 2016–2018 window represents a concentrated emergence of core literature, with pivotal papers like Gulshan et al. (9) and He et al. (29) serving as critical bridges linking general technologies to clinical applications. Looking ahead, emerging frontiers include multimodal fusion, cross-ethnicity and cross-device generalizability/fairness, and the integration of foundation models and large vision-language models. However, literature accumulation for these new paradigms remains nascent, and substantive structural transformation warrants continued observation.

### 4.3 Evolutionary trajectory of research hotspots

Keyword co-occurrence maps (**Figure 7**) delineate a progressive research chain spanning “disease quantification → feature extraction → classification/segmentation → clinical validation → multi-disease expansion”. “Accuracy” and “classification” emerge as pivotal hubs linking technological development to clinical utility, reinforcing the consensus that algorithmic advances must be paralleled by rigorous clinical evaluation. Prior to 2004, studies relied predominantly on conventional computer vision for basic vessel segmentation (50). Between 2015 and 2017, CNNs triggered a research surge, with emphasis shifting rapidly toward diagnostic accuracy (51). The recent distribution of keywords (**Figure 7C**), however, reveals a decisive turn: the field is pivoting from pure algorithmic refinement toward model interpretability (XAI), cross-modal data fusion, and real-world deployment in primary care (52, 53). Notably, concepts such as “explainability” are transitioning from peripheral topics to central research themes, signaling that future AI-driven DR research will prioritize tangible clinical utilities – such as predicting systemic microvascular complications from fundus imaging and enhancing model robustness against complex pathological phenotypes (54, 55).

### 4.4 Clinical needs and technological maturation

The evolution of data modalities reflects a synergy between clinical imperatives and technological readiness. While early investigations depended heavily on static fundus photography (56), contemporary research leverages OCT volumetric analytics (57), OCTA flow-density metrics (58), and DL-enabled visual field interpretation to drive a shift toward objective, quantitative endpoints. The ubiquity of standardized digital imaging – particularly fundus photography and OCT – has furnished large-scale, high-fidelity datasets for AI training. Visual field testing remains indispensable for Glaucoma surveillance, where longitudinal monitoring of functional deterioration generates extensive clinical datasets. OCT, often described as a revolutionary advancement in ophthalmic imaging, permits precise quantification of microstructural alterations, such as retinal nerve fiber layer thinning. OCTA has recently revolutionized non-invasive angiography; its high-resolution, dye-free visualization of the retinal vasculature has rapidly established it as a hotspot for investigating diabetic retinopathy and AMD (59). Concurrently, the accrual of genomic data heralds the advent of precision ophthalmology (60, 61). For genetically predisposed conditions like *Primary Open-Angle Glaucoma* (POAG) and AMD, gene discovery and molecular phenotyping are becoming integral to elucidating pathogenesis and tailoring individualized therapies.

### 4.5 Associations among AI models, data modalities, and diseases

The interplay among AI models, data modalities, and diseases is not merely a descriptive observation but a fundamental organizing principle governing the field’s evolution. To systematically unpack this coupling, we approach it from multiple angles. These perspectives collectively reveal how algorithmic choices, data availability, and disease targets co-evolve and reinforce one another. To ground this multi-angle analysis in concrete evidence, **Table 3** lists the principal AI models that have been applied to diabetic retinopathy, summarizing their publication years, typical data modalities, primary tasks, and key strengths and limitations. This inventory not only facilitates cross-model comparison but also serves as a reference point for the temporal and functional trends elaborated in the following subsections.

**Table 3:** Summary of the mainstream models for DR analysis.

Model	Original year	Data modality	Main tasks in DR	Advantages	Disadvantages
Transformer	Vaswani et al. (62)	Fundus images, OCT, EHR data	Image classification, grading, risk prediction	Powerful global feature capture; strong performance	High computational cost; requires large datasets
ViT	Dosovitskiy et al. (63)	Color fundus photos, OCT	DR grading, detection	Scalable; performance improves with model size	Computationally heavy; less precise for small lesion localization
CNN	LeCun et al. (64)	Fundus images, OCT	Lesion detection, segmentation, severity assessment	Mature technology; wide applicability; good performance	Limited generalization; poor interpretability
GAN	Goodfellow et al. (65)	Fundus images	Image generation and augmentation	Generates realistic images; augments small datasets	Challenges in fine detail generation; training instability
ResNet	He et al (29)	Fundus images	Image classification, severity grading	Solves vanishing gradients via skip connections; enables deep networks	Slow convergence; Heavy parameter tuning
U-Net	Ronneberger et al. (30)	Fundus images, OCT	Image segmentation (retinal layers, lesions)	Classic baseline for medical image segmentation	Performance drops on complex pathologies
SVM	Cortes and Vapnik (66)	Clinical data, handcrafted image features	Classification, microaneurysm detection,risk prediction	Robust on small datasets; relatively interpretable	Sensitive to image noise; underperforms on large-scale image data
Logistic Regression	LaValley (67)	Clinical data (e.g., HbA1c)	Risk prediction, disease classification	Simple, highly interpretable, less overfitting	Limited ability to handle complex nonlinear relationships
Inception	Szegedy et al. (68)	Fundus images	Image classification, DR grading	Strong multi-scale feature extraction	May fail on the most challenging cases
VGG	Simonyan and Zisserman (31)	Fundus images	Image classification, severity grading	Simple architecture, reliable performance	High computational resource demand
DenseNet	Huang et al. (69)	Fundus images, OCT	Image classification, severity grading	Excellent performance; parameter-efficient	High memory consumption as network deepens
EfficientNet	Tan and Le (70)	Fundus images	Image classification, DR grading	State-of-the-art performance; high efficiency	Risk of overfitting; limited performance on severe grades
Random Forest	Breiman (71)	Clinical data, handcrafted features	Classification, risk prediction	Robust to noise; good interpretability	Requires manual feature engineering
MobileNet	Howard et al. (72)	Fundus images	Image classification, disease grading	Lightweight architecture; suitable for mobile real-time use	May sacrifice accuracy for speed
Decision Tree	Stone et al. (73)	Clinical data	Risk prediction, disease classification	Very simple, fully interpretable	Prone to overfitting; low stability

Years denote when the model was first proposed in computer vision/ML; its adoption for DR typically came later.

#### 4.5.1 Catalysts of the publication surge: synergy between algorithmic evolution and screening imperatives

The exponential growth in publications post-2010 (**Figures 8**-**10**) stems primarily from three convergent factors. First, the maturation of DL – exemplified by the transition from early modern CNNs (AlexNet, 2012) to deeper architectures such as VGG, Inception, ResNet, and DenseNet – has enabled direct training on fundus and OCT datasets for lesion segmentation, disease grading, and screening (74, 75). The proliferation of public datasets and open-source frameworks has markedly lowered entry barriers, fueling the expansion of imaging-related literature. Second, global initiatives for DR and AMD screening have generated massive repositories of fundus and OCT data, facilitating epidemiological modeling and large-cohort studies. While CNNs have long anchored due to their local receptive fields and parameter sharing – which are inherently suited to 2D fundus texture and edge features, with VGG offering reliable simplicity and DenseNet providing parameter-efficient performance – these architectures are not without constraints. EfficientNet, despite achieving state-of-the-art efficiency via compound scaling, often exhibits overfitting on severe disease grades due to class imbalance (8). Recently, however, the increasing publication volume of ViT and Transformer architectures reflects a quest for global context; ViTs capture long-range spatial dependencies across entire fundus fields or OCT volumes, enhancing the detection of subtle, dispersed lesions (76), though this gain comes at the cost of substantially higher data and computational demands compared to lightweight alternatives like MobileNet, which prioritizes on-device deployment efficiency.

#### 4.5.2 Differential adoption of modalities: clinical utility and data characteristics

Disparities in publication volume across modalities mirror underlying clinical and logistical determinants. Fundus photography dominates due to its cost-effectiveness, scalability, and archival simplicity, aligning seamlessly with public health screening mandates. OCT retains centrality by enabling quantitative morphometry – measuring layer thickness, edema volume, and exudate burden – critical for mechanistic studies and therapeutic monitoring. Conversely, FA, being invasive, is largely confined to adjudicating diagnostically challenging cases (77), with its niche progressively encroached upon by non-invasive OCTA. Genomics, focused on monogenic disorders such as Retinitis Pigmentosa, is experiencing steady growth as sequencing costs decline, underpinning familial studies and susceptibility locus mapping (78). Functional tests (visual fields, ERG) primarily serve as adjunctive outcome measures complementing structural imaging. Crucially, beyond imaging, traditional machine learning models – including SVM, Random Forest, and Logistic Regression – persist in parallel niches, primarily handling structured clinical tabular data (e.g., HbA1c levels) (79). Their enduring presence is attributable to high interpretability and robustness on small cohorts.

#### 4.5.3 Architectural specialization and the rise of multimodal integration

Distinct architectural paradigms exhibit functional specialization in DR management. For disease grading (e.g., per ETDRS scales), architectures like ViT, Transformers, and ResNet classify severity by parsing global patterns; ViT’s patch-based attention mechanism has shown promising performance in retrospective research settings (80). Inception’s multi-scale convolutional kernels further enhance the detection of variably sized lesions, though they may fail on the most diagnostically challenging presentations (81). U-Net and its variants sustain robust growth, reflecting the premium placed on pixel-level lesion localization (82). Its encoder-decoder architecture preserves fine-grained edge details, providing quantitative morphometric evidence for precise localization of focal lesions such as microaneurysms and hard exudates, directly supporting individualized treatment planning (83). Nevertheless, U-Net’s performance may degrade when confronting highly complex pathological morphologies, necessitating architectural enhancements.

Recognizing the inherent limitations of standalone color fundus photography, frontier research is integrating multimodal data – combining OCT’s structural insights with OCTA’s angiographic perfusion metrics – to holistically assess retinal ischemia and microcirculatory dysfunction, thereby refining progression risk stratification. Furthermore, the persistent presence of *Generative Adversarial Networks* (GANs) (**Figures 11**, **12**) underscores their utility in synthesizing high-fidelity pathological imagery to augment scarce annotated datasets, bolstering model generalization to rare, severe phenotypes (84), although their training instability and difficulty in generating fine lesion details remain active areas of methodological refinement. More profoundly, the emergence of multimodal *Large Language Models* (LLMs) portends a paradigm shift. Vision-language models, exemplified by DeepDR-LLM (85), transcend mere image classification; they synthesize multimodal inputs – including clinical history and medication records – to generate personalized management recommendations (86). This evolution suggests a potential trajectory of AI-driven DR care from “single-disease pattern recognition” toward “multidimensional prognostic assessment and comprehensive longitudinal disease management”. Genomics, though captured in our co-occurrence maps, remains limited in DR-specific literature. In the DR context, gene therapy studies primarily focus on inflammatory pathways, representing no departure from the core topics of DR research. It is important to note that current evidence remains largely exploratory.

### 4.6 Current limitations and future directions

#### 4.6.1 Existing bottlenecks

Despite substantial progress in AI-driven DR research, translation from bench to bedside remains constrained by several structural barriers.

##### 4.6.1.1 Data silos and under-integrated multimodality

Imaging, genomics, and *Electronic Health Records* (EHRs) are typically housed in separate database systems, and paired cross-modal datasets are exceedingly scarce, leaving integrated imaging-genomics-clinical-text analyses underrepresented. Moreover, systemic imaging modalities such as ultrasound remain underutilized in ophthalmology and are seldom used to investigate systemic associations between fundus pathology and neurologic disease or microvascular pathology (87). Meanwhile, clinical free text poses substantial structuring challenges; EHR-mining publications significantly trail imaging-based studies, leaving diagnostically rich patient records largely untapped (88).

##### 4.6.1.2 Rare-disease evidence gaps and limited omics-imaging integration

For rare fundus disorders such as Best Disease and specific hereditary retinal degenerations, limited sample sizes preclude large-scale cohort work, confining most studies to mechanistic exploration (89). Long-term follow-up, health-economic evaluation, and community-screening infrastructure constitute a persistently small fraction of the literature, constraining the translational impact of research on public-health policy. Furthermore, multi-omics data (transcriptomics, metabolomics) are rarely combined with imaging, hindering molecular subtyping and precise phenotype-genotype correlation.

##### 4.6.1.3 Deployment barriers and portable acquisition

Real-world device compatibility, data heterogeneity, and cross-population generalizability remain insufficiently validated (90). Community-based screening and home-monitoring infrastructures are underdeveloped, and the potential of lightweight, portable devices (e.g., smartphone-based fundus cameras) has not been systematically explored in research settings, limiting the collection of diverse, real-world datasets and the feasibility of decentralized screening models.

##### 4.6.1.4 Translational closure and explainable AI

A marked gap persists between technical maturity and clinical adoption. External validation lacks standardized protocols, and performance variability across datasets remains incompletely characterized (91). Moreover, integration of AI systems into clinical workflows, health-economic assessment, and regulatory pathways remains largely exploratory, with no mature closed-loop translation mechanism yet in place. In addition, current “black-box” models offer limited interpretability, which undermines clinician trust and impedes adoption in routine practice, especially in sight-threatening clinical decisions.

#### 4.6.2 Future trajectory and research roadmap

Future AI-driven DR research is likely to coalesce around four axes: multimodal fusion, omics-imaging integration, portable platforms, and explainable AI. However, as these projections are extrapolated from current literature trends, they should be interpreted as directional indicators.

##### 4.6.2.1 Multimodal fusion and foundation-model development

The core paradigm of future research could shift from single-modality analysis (e.g., fundus photography or OCT alone) toward a multimodal integration framework that systematically incorporates heterogeneous data sources – including structural imaging, angiography, visual field, genomics, and clinical text. A unified ophthalmic foundation model appears to be a priority in this trajectory. Through cross-modal transfer learning, such models can capture shared disease representations across disparate data views, substantially improving differential diagnostic capability and subtype discrimination for complex cases (92). At the translation level, research outputs may increasingly translate from publications into deployable clinical decision-support tools, propelling AI systems from laboratory validation into real-world workflows – spanning screening and early warning, assisted diagnosis, treatment planning, and prognostic prediction. Concurrently, the research scope could expand from single-disease analysis toward multi-disease comorbidity modeling. Complex scenarios – such as diabetic retinopathy complicated by hypertensive retinopathy, or multiple ocular conditions co-existing in aging populations – could constitute core subjects of future work, more faithfully reflecting the full disease profile of real-world patients and enhancing the clinical applicability and decision-making value of AI models (93).

##### 4.6.2.2 Deep omics-imaging integration and precision subtyping

Multi-omics joint analysis – integrating transcriptomics, metabolomics, and fundus imaging – could transcend current morphology-only phenotypic limits, offering new leverage for molecular subtyping, prognostic prediction, and individualized therapy for retinal disease, and narrowing the gap between basic science and clinical phenotype (94, 95). Gene-therapy trials for Retinitis Pigmentosa and selected hereditary macular dystrophies are advancing steadily and are likely to catalyze a new wave of studies in rare ocular disease, with associated genetic-testing and efficacy-assessment work becoming an important growth pole in basic ophthalmic research.

##### 4.6.2.3 Portable acquisition platforms as new research streams

Lightweight portable devices, such as smartphone-based fundus cameras, will create entirely new research streams, enabling image acquisition beyond the ophthalmic clinic and generating substantive work in home-based monitoring, community screening, and tele-ophthalmology – driving DR screening from a centralized toward a decentralized model (96).

##### 4.6.2.4 Explainable AI to foster clinical trust

Current “black-box” models (e.g., ViT, ResNet) still struggle with data scarcity in rare-disease recognition. A key advance involves adopting XAI frameworks that output not only a diagnosis (“what”) but also a rationale (“why”) via lesion-focused saliency maps and transparent reasoning paths – potentially propelling ophthalmic AI from assisted screening toward deep-level decision support (97).

## 5 Limitations of the study and future work

When interpreting the findings of this study, the following limitations must be fully considered. These limitations primarily stem from the inherent nature of bibliometric methods and affect only the granularity of quantitative results, without undermining the core conclusions demonstrated in this study – namely, the three-stage evolutionary trajectory of the field, the multipolar pattern of global collaboration, and the profound interdependencies among algorithms, data, and diseases.

### 5.1 Data sources, time frame, and representativeness

This study analyzed English-language literature indexed in three major databases from January 1996 to June 2026. Notably, publication counts primarily reflect the macro-level distribution of research activity and are not necessarily directly equivalent to clinical translational impact. The starting year of 1996 was chosen with the aim of covering a substantial portion of the evolutionary arc of AI in medicine – from the early incorporation of machine learning into medical informatics, through the deep learning breakthrough marked by AlexNet in 2012, to the more recent rise of large language models and multimodal systems – thereby enabling a more detailed mapping of the trajectory from theoretical exploration to clinical application in DR. Nevertheless, the following inherent biases remain:

Database coverage bias: Although the three databases predominantly index high-quality peer-reviewed literature, they inherently exclude certain preprints (e.g., arXiv, bioRxiv, medRxiv) and regional non-indexed journals. Moreover, indexing criteria vary across platforms, and our PubMed retrieval strategy – restricting searches to the title field only– further limits recall range: studies in which core terms appear solely in the abstract or main text may have been omitted. This study mitigated this limitation through cross-database stratified deduplication and pre-validation of search strategies; however, complete elimination of this coverage bias remains infeasible.

Citation delay bias: Bibliometric analyses are subject to a publication-citation time lag. Studies published in recent years have not yet had sufficient time to accrue citations, which may lead to an underestimation of emerging hotspots (e.g., ophthalmology-specific foundation models, multimodal alignment techniques) in burst detection analyses and impact assessments.

Self-citation bias: This study did not apply specific corrections for self-citations. Highly productive authors or institutions may artificially inflate citation counts and network centrality metrics through excessive self-citation, potentially inflating the perceived academic influence of certain research teams. Furthermore, dense internal citations within core research clusters may disproportionately highlight mainstream technological pathways while marginalizing niche yet promising directions. Future analyses should incorporate self-citation rate adjustments to enhance objectivity.

Indexing bias: The search strategy relied predominantly on general AI terminology and did not explicitly include emerging specialized terms such as “foundation models”, “vision-language models”, or “self-supervised learning”. Although the vast majority of cutting-edge literature retains general terms in titles, abstracts, and keywords to maximize discoverability, some purely applied studies may have been missed. This likely introduces bibliometric discrepancies, particularly in the later stages of the study. Therefore, the results presented herein should be regarded as indicative metrics of overall research vitality and developmental trends in the AI field, rather than as an exhaustive inventory of all literature pertaining to every emerging derivative term.

Rapid-iteration literature bias: The AI field evolves rapidly, with short iteration cycles spanning algorithmic proposal, validation, and publication. The retrieval cutoff date of June 2026 implies that frontier paradigms emerging between late 2025 and the first half of 2026 (e.g., ophthalmology-specific foundation models, federated learning-based cross-center screening frameworks) may not have completed peer review or been formally indexed, resulting in a temporal lag in capturing the latest technological trends. Consequently, readers should interpret conclusions regarding frontier trajectories in conjunction with recent preprints and industry developments.

### 5.2 Language restriction and potential geographic bias

The restriction to English-language literature imposes constraints on the global perspective, manifesting in two primary aspects:

Underestimation of regional contributions: The omission of non-English literature may result in insufficient coverage of large-scale screening studies conducted on local populations in regions such as East Asia, Latin America, and Eastern Europe, thereby partially underestimating the contributions of these regions to the field.

Incomplete visibility of collaborations: The international collaboration networks mapped in this study reflect only partnerships disseminated through English-language journals. If the outputs of regional research consortia are not published in internationally accessible outlets, their collaborative patterns will remain undetected in our analysis.

Notably, while these limitations affect the granularity of the global landscape, they are unlikely to invalidate the core developmental patterns and principal conclusions derived from this study.

### 5.3 Limitations of the rule-based keyword classification approach

Although the rule-based keyword classification approach adopted in this study strives to cover a broad spectrum of architectures, data modalities, and disease entities – ranging from traditional machine learning to ophthalmic foundation models – several limitations remain.

Dictionary coverage is inherently incomplete. Despite the inclusion of numerous emerging architectures and their variants, it remains difficult in practice to exhaustively enumerate all existing model types, including those rapidly evolving. Models not present in the dictionary either remain unrecognised or are erroneously assigned to a broader parent category, which may lead to underestimation of novel architectural trends and overestimation of the relative share of generic categories.

Text mining introduces inherent biases. Our analysis relies solely on titles, abstracts, and keyword fields; consequently, any mention of models or diseases appearing exclusively in the full text or appendices is not captured. Moreover, the mere presence of a model name or disease term does not necessarily indicate that the mentioned model or disease constitutes the core methodology or primary research focus of the paper. Consequently, the quantitative trends presented here primarily reflect the scholarly attention paid to specific topics within the field, rather than their actual clinical deployment frequency or technological dominance.

Co-occurrence does not imply causation. In addition, given the relatively short observation window for several recently proposed ophthalmic foundation models, a comprehensive assessment of their long-term evolutionary trajectories is constrained. Future work should continuously update the dictionaries, expand data sources, and adopt semantic embedding models to mitigate systematic biases arising from strict lexical boundaries, thereby enhancing both the depth and breadth of such analyses.

### 5.4 Evidence boundary for emerging technical claims

A fundamental limitation of bibliometric analysis is that publication frequency reflects research activity and scholarly interest, rather than clinical efficacy, algorithmic superiority, or real-world adoption. Consequently, the observed prominence of specific architectures reflects a significant increase in academic attention rather than demonstrated clinical utility. Since this study did not conduct performance benchmarking against established baselines or incorporate metrics of clinical translation, our inferences solely characterize the shift in research hotspots. These findings should not be construed as evidence of definitive technical superiority or translational maturity.

### 5.5 Future work

To mitigate the structural biases arising from data source constraints, language filtering, and uncorrected self-citations, future research should advance in the following directions:

Integration of multi-source heterogeneous data: Subsequent studies should draw upon outputs from preprint platforms and strategically include high-value regional non-English journals to construct a multi-source data fusion framework. This would enable higher-resolution tracking of algorithmic innovations and clinical translation dynamics in global AI-driven DR diagnosis and care. Concurrently, self-citation rate correction protocols should be implemented, and cross-database indexing discrepancies should be harmonized to improve the objectivity of quantitative indicators.

Analysis of dynamic evolutionary mechanisms: Dynamic topic modeling and automated clustering methodologies should be employed to distinguish transient research hotspots from substantive foundational contributions, thereby constructing a full-chain evolutionary framework bridging theoretical algorithmic breakthroughs with clinical deployment. This approach would account for the rapid-iteration nature of AI literature and provide a methodological basis for longitudinal updates.

## 6 Conclusion

This study systematically delineates the developmental trajectory, knowledge architecture, hotspot migration trends, and dynamic high-frequency co-occurrence mechanisms of AI in DR research over the past three decades (1996-2026).

The core findings are summarized below.

First, the field has followed a distinct three-stage trajectory: an early phase anchored traditional machine learning and handcrafted feature engineering; a middle phase driven by CNNs that established the end-to-end learning paradigm; and the current phase marked by rapidly growing research attention to ViTs and large-scale vision models, which prioritize global contextual modeling and multimodal integration as an exploratory direction. This evolution suggests potential for further gains in diagnostic accuracy, model robustness, and generalization. Notably, high-frequency co-occurrence patterns are observed among AI model families, data modalities, and diseases. CNNs–exemplified by ResNet and U-Net – have long constituted the mainstream in DR classification and segmentation based on fundus photography and OCT. Recent publication trends reflect growing interest in ViT-based, fundus-OCT multimodal frameworks for broader retinal disease phenotyping, though their long-term sustainability and clinical utility require further validation.

Second, the global research ecosystem exhibits a multipolar but stratified and asymmetrical pattern. The United States, China, and India constitute the primary output hubs, with the Asia-Pacific functioning as both a core research region and a cluster of high-impact contributions. Singapore, leveraging tightly integrated clinical-academic-industry clusters, has emerged as a pivotal node in international collaboration. Nevertheless, disparities persist between publication volume and citation impact.

Third, the field’s knowledge architecture displays a dual-core coupling structure driven by engineering-technological innovation and supported by clinical validation. Data modalities have evolved from single fundus photography toward multimodal integration encompassing OCT, OCTA, genomics, and EHR-derived data; application scenarios have expanded from DR screening alone to broader retinal disease management and prediction of systemic microvascular complications.

Fourth, the knowledge base rests on two interdependent pillars: clinical validation studies in high-impact medical journals, and foundational algorithmic contributions in engineering and computer science. Co-citation and keyword networks trace a knowledge-flow chain – from disease burden assessment and image processing through deep feature extraction and large-scale clinical validation to model interpretability and real-world deployment – signaling a field-wide shift from “black-box” pattern recognition toward trustworthy AI and the full care continuum.

Despite remarkable progress, persistent bottlenecks remain: entrenched data silos, underutilization of non-imaging data, limited evidence on rare DR subtypes and long-tail phenotypes, and a translational gap between technical performance and clinical workflow integration.

Future research should prioritize unified ophthalmic foundation models that integrate imaging, genomics, and clinical text to enable precise subtype stratification and personalized management. Concurrently, the proliferation of portable smartphone-based fundus devices and advances in explainable AI will be critical to lowering technical barriers and fostering clinical adoption.

## Data Availability

The original contributions presented in the study are included in the article/**Supplementary Material**. Further inquiries can be directed to the corresponding author.

## References

[1] Li H Liu X Zhong H Fang J Li X Shi R . Research progress on the pathogenesis of diabetic retinopathy. BMC Ophthalmol. (2023) 23:372. doi: 10.1186/s12886-023-03118-6 37697295 PMC10494348

[2] Irodi A Zhu Z Grzybowski A Wu Y Cheung CY Li H . The evolution of diabetic retinopathy screening. Eye. (2025) 39:1040–6. doi: 10.1038/s41433-025-03633-4 39910282 PMC11978858

[3] Kumar V Paul K . Fundus imaging-based healthcare: Present and future. ACM Trans Comput Healthcare. (2023) 4:1–34. doi: 10.1145/3586580

[4] Tan TF Thirunavukarasu AJ Jin L Lim J Poh S Teo ZL . Artificial intelligence and digital health in global eye health: opportunities and challenges. Lancet Global Health. (2023) 11:e1432–43. doi: 10.1016/s2214-109x(23)00323-6 37591589

[5] Grzybowski A Brona P Lim G Ruamviboonsuk P Tan GS Abramoff M . Artificial intelligence for diabetic retinopathy screening: a review. Eye. (2020) 34:451–60. doi: 10.1038/s41433-019-0566-0 31488886 PMC7055592

[6] Imran A Li J Pei Y Yang J-J Wang Q . Comparative analysis of vessel segmentation techniques in retinal images. IEEE Access. (2019) 7:114862–87. doi: 10.1109/access.2019.293591225079929

[7] Muthusamy D Palani P . Deep learning model using classification for diabetic retinopathy detection: an overview. Artif Intell Rev. (2024) 57:1. doi: 10.1007/s10462-024-10806-230311153

[8] Arora L Singh SK Kumar S Gupta H Alhalabi W Arya V . Ensemble deep learning and efficientnet for accurate diagnosis of diabetic retinopathy. Sci Rep. (2024) 14:30554. doi: 10.1038/s41598-024-81132-4 39695310 PMC11655640

[9] Gulshan V Peng L Coram M Stumpe MC Wu D Narayanaswamy A . Development and validation of a deep learning algorithm for detection of diabetic retinopathy in retinal fundus photographs. Jama. (2016) 316:2402–10. doi: 10.1001/jama.2016.17216 27898976

[10] Raja H Akram MU Shaukat A Khan SA Alghamdi N Khawaja SG . Extraction of retinal layers through convolution neural network (CNN) in an OCT image for glaucoma diagnosis. J Digital Imaging. (2020) 33:1428–42. doi: 10.1007/s10278-020-00383-5 32968881 PMC7728844

[11] Liu X Zhang D Yao J Tang J . Transformer and convolutional based dual branch network for retinal vessel segmentation in OCTA images. BioMed Signal Process Control. (2023) 83:104604. doi: 10.1016/j.bspc.2023.10460442574925

[12] Wan S Liang Y Zhang Y . Deep convolutional neural networks for diabetic retinopathy detection by image classification. Comput Electr Eng. (2018) 72:274–82. doi: 10.1016/j.compeleceng.2018.07.04242574925

[13] Sambyal N Saini P Syal R Gupta V . Modified u-net architecture for semantic segmentation of diabetic retinopathy images. Biocybern BioMed Eng. (2020) 40:1094–109. doi: 10.1016/j.bbe.2020.05.00642574925

[14] Kanclerz P Tuuminen R Khoramnia R . Imaging modalities employed in diabetic retinopathy screening: a review and meta-analysis. Diagnostics. (2021) 11:1802. doi: 10.3390/diagnostics11101802 34679501 PMC8535170

[15] Rajalakshmi R Subashini R Anjana RM Mohan V . Automated diabetic retinopathy detection in smartphone-based fundus photography using artificial intelligence. Eye. (2018) 32:1138–44. doi: 10.1038/s41433-018-0064-9 29520050 PMC5997766

[16] Zhao R Gillani S . AI in ophthalmology: A bibliometric analysis of retinal imaging innovations and global research collaboration. Photodiagn Photodyn Ther. (2026) 59:105458. doi: 10.1016/j.pdpdt.2026.105458 41932561

[17] Poly TN Islam MM Walther BA Lin MC Li Y-CJ . Artificial intelligence in diabetic retinopathy: bibliometric analysis. Comput Methods Programs BioMed. (2023) 231:107358. doi: 10.1016/j.cmpb.2023.107358 36731310

[18] Shao A Jin K Li Y Lou L Zhou W Ye J . Overview of global publications on machine learning in diabetic retinopathy from 2011 to 2021: bibliometric analysis. Front Endocrinol. (2022) 13:1032144. doi: 10.3389/fendo.2022.1032144 36589855 PMC9797582

[19] Ghazali AFB . A scientometric analysis and visualisation of research on deep learning for diabetic retinopathy. In: 2025 International Conference on Advances in Machine Intelligence, and Cybersecurity Technologies (AMICT). Piscataway, New Jersey: IEEE (2025). p. 174–9. doi: 10.1109/AMICT65811.2025.11402804

[20] Huang Y Qi Y Liu C Jing F Li C Wang M . A decade of progress in artificial intelligence for fundus image-based diabetic retinopathy screening, (2014–2024): a bibliometric analysisA decade of progress in artificial intelligence for fundus image-based diabetic retinopathy screening, (2014–2024): a bibliometric analysis. MedRxiv. (2024), 2024–11. doi: 10.1101/2024.11.02.2431663538621210

[21] Lin M Lin L Lin L Lin Z Yan X . A bibliometric analysis of the advance of artificial intelligence in medicine. Front Med. (2025) 12:1504428. doi: 10.3389/fmed.2025.1504428 40061376 PMC11885233

[22] Li M Chen S Liu S Yang J Qin Y Chen Y . A bibliometric analysis of the global research landscape on artificial intelligence applications in clinical medicine, (2010–2025). DIGITAL Health. (2026) 12:20552076261443381. doi: 10.1177/20552076261443381 41993044 PMC13080151

[23] Alotaibi A Contreras R Thakker N Mahapatro A Adla Jala SR Mohanty E . Bibliometric analysis of artificial intelligence applications in cardiovascular imaging: trends, impact, and emerging research areas. Ann Med Surg. (2025) 87:1947–68. doi: 10.1097/ms9.0000000000003080 40212204 PMC11981274

[24] Ting DSW Cheung CY-L Lim G Tan GSW Quang ND Gan A . Development and validation of a deep learning system for diabetic retinopathy and related eye diseases using retinal images from multiethnic populations with diabetes. Jama. (2017) 318:2211–23. doi: 10.1001/jama.2017.18152 29234807 PMC5820739

[25] Gargeya R Leng T . Automated identification of diabetic retinopathy using deep learning. Ophthalmology. (2017) 124:962–9. doi: 10.1016/j.ophtha.2017.02.008 28359545

[26] Abràmoff MD Lou Y Erginay A Clarida W Amelon R Folk JC . Improved automated detection of diabetic retinopathy on a publicly available dataset through integration of deep learning. Invest Ophthalmol Visual Sci. (2016) 57:5200–6. doi: 10.1167/iovs.16-19964 27701631

[27] Abràmoff MD Lavin PT Birch M Shah N Folk JC . Pivotal trial of an autonomous AI-based diagnostic system for detection of diabetic retinopathy in primary care offices. NPJ Digital Med. (2018) 1:39. doi: 10.1038/s41746-018-0040-6 PMC6550188 31304320

[28] LeCun Y Bengio Y Hinton G . Deep learning. Nature. (2015) 521:436–44. doi: 10.1038/nature14539 26017442

[29] He K Zhang X Ren S Sun J . (2016). Deep residual learning for image recognition. In: Proceedings of the IEEE Conference on Computer Vision and Pattern Recognition (Piscataway, New Jersey: IEEE) p. 770–8.

[30] Ronneberger O Fischer P Brox T . (2015). “ U-net: Convolutional networks for biomedical image segmentation”, in: International Conference on Medical Image Computing and Computer-Assisted Intervention (MICCAI) (Cham: Springer) 9352:234–41. doi: 10.1007/978-3-319-24574-4_28

[31] Simonyan K Zisserman A . Very deep convolutional networks for large-scale image recognition. ArXiv Preprint ArXiv:14091556. (2014). doi: 10.48550/arXiv.1409.1556

[32] Szegedy C Vanhoucke V Ioffe S Shlens J Wojna Z . (2016). “ Rethinking the inception architecture for computer vision.” In: Proceedings of the IEEE Conference on Computer Vision and Pattern Recognition. (Las Vegas, NV: IEEE), p. 2818–26.

[33] Staal J Abràmoff MD Niemeijer M Viergever MA Van Ginneken B . Ridge-based vessel segmentation in color images of the retina. IEEE Trans Med Imaging. (2004) 23:501–9. doi: 10.1109/tmi.2004.825627 15084075

[34] Hoover A Kouznetsova V Goldbaum M . Locating blood vessels in retinal images by piecewise threshold probing of a matched filter response. IEEE Trans Med Imaging. (2000) 19:203–10. doi: 10.1109/42.845178 10875704

[35] Decencière E Zhang X Cazuguel G Lay B Cochener B Trone C . Feedback on a publicly distributed image database: the messidor database. Image Anal Stereol. (2014) 231–4. doi: 10.5566/ias.1155

[36] Quellec G Charriere K Boudi Y Cochener B Lamard M . Deep image mining for diabetic retinopathy screening. Med Image Anal. (2017) 39:178–93. doi: 10.1016/j.media.2017.04.012 28511066

[37] Porwal P Pachade S Kamble R Kokare M Deshmukh G Sahasrabuddhe V . Indian diabetic retinopathy image dataset (idrid): a database for diabetic retinopathy screening research. Data. (2018) 3:25. doi: 10.3390/data3030025

[38] Li T Gao Y Wang K Guo S Liu H Kang H . Diagnostic assessment of deep learning algorithms for diabetic retinopathy screening. Inf Sci. (2019) 501:511–22. doi: 10.1016/j.ins.2019.06.011

[39] Wilkinson CP Ferris FL III Klein RE Lee PP Agardh CD Davis M . Proposed international clinical diabetic retinopathy and diabetic macular edema disease severity scales. Ophthalmology. (2003) 110:1677–82. doi: 10.1016/S0161-6420(03)00475-5 13129861

[40] Yau JW Rogers SL Kawasaki R Lamoureux EL Kowalski JW Bek T . Global prevalence and major risk factors of diabetic retinopathy. Diabetes Care. (2012) 35:556–64. doi: 10.2337/dc11-1909 PMC3322721 22301125

[41] Kermany DS Goldbaum M Cai W Valentim CC Liang H Baxter SL . Identifying medical diagnoses and treatable diseases by image-based deep learning. Cell. (2018) 172:1122–31. doi: 10.1016/j.cell.2018.02.010 29474911

[42] De Fauw J Ledsam JR Romera-Paredes B Nikolov S Tomasev N Blackwell S . Clinically applicable deep learning for diagnosis and referral in retinal disease. Nat Med. (2018) 24:1342–50. doi: 10.1038/s41591-018-0107-6 30104768

[43] Korot E Gonçalves MB Huemer J Beqiri S Khalid H Kelly M . Clinician-driven AI:code-free self-training on public data for diabetic retinopathy referral. JAMA Ophthalmol. (2023) 141:1029–36. doi: 10.1001/jamaophthalmol.2023.4508 37856110 PMC10587830

[44] Li D Huang R Cui C Towey D Zhou L Tian J . Ribonucleic-acid protein interaction prediction based on deep learning: A comprehensive survey. Appl Soft Comput. (2025) 184:113795. doi: 10.1016/j.asoc.2025.11379542574925

[45] Shakor MY Khaleel MI . Recent advances in big medical image data analysis through deep learning and cloud computing. Electronics. (2024) 13:4860. doi: 10.3390/electronics1324486030654563

[46] Kodani N Kato A Lee M-K Ma RCW Sabidi A Scibilia R . Diabetes advocacy in the asia–pacific region. J Diabetes Invest. (2025) 16:1191–201. doi: 10.1111/jdi.70084 40421836 PMC12209509

[47] Duggal M Chauhan A Gupta V Kankaria A Budhija D Verma P . Real-world evaluation of AI-driven diabetic retinopathy screening in public health settings: Validation and implementation study. JMIR Med Inf. (2025) 13:e67529. doi: 10.2196/67529 40925861 PMC12419978

[48] Quellec G Lamard M Cochener B Decencière E Lay B Chabouis A . (2013). “ Multimedia data mining for automatic diabetic retinopathy screening”, in: 2013 35th Annual International Conference of the IEEE Engineering in Medicine and Biology Society (EMBC) (Piscataway, New Jersey: IEEE), 7144–7. 10.1109/EMBC.2013.6611205 24111392

[49] Zhang X Thibault G Decencière E Marcotegui B Laÿ B Danno R . Exudate detection in color retinal images for mass screening of diabetic retinopathy. Med Image Anal. (2014) 18:1026–43. doi: 10.1016/j.media.2014.05.004 24972380

[50] Teng T Lefley M Claremont D . Progress towards automated diabetic ocular screening: a review of image analysis and intelligent systems for diabetic retinopathy. Med Biol Eng Comput. (2002) 40:2–13. doi: 10.1007/bf02347689 11954703

[51] Pratt H Coenen F Broadbent DM Harding SP Zheng Y . Convolutional neural networks for diabetic retinopathy. Proc Comput Sci. (2016) 90:200–5. doi: 10.1016/j.procs.2016.07.01442574925

[52] Lim WX Chen Z Ahmed A . The adoption of deep learning interpretability techniques on diabetic retinopathy analysis: a review. Med Biol Eng Comput. (2022) 60:633–42. doi: 10.1007/s11517-021-02487-8 35083634 PMC8791699

[53] Qin G Xu N Xu J . (2025). “ Multimodal deep learning for diabetic retinopathy: A survey”, in: 2025 IEEE 8th International Conference on Multimedia Information Processing and Retrieval (MIPR) (Piscataway, New Jersey: IEEE), 151–7.

[54] Tan YY Kang HG Lee CJ Kim SS Park S Thakur S . Prognostic potentials of AI in ophthalmology: systemic disease forecasting via retinal imaging. Eye Vision. (2024) 11:17. doi: 10.1186/s40662-024-00384-3 38711111 PMC11071258

[55] Yang Q Bee YM Lim CC Sabanayagam C Cheung CY-L Wong TY . Use of artificial intelligence with retinal imaging in screening for diabetes-associated complications: systematic review. EClinicalMedicine. (2025) 81:103089. doi: 10.1016/j.eclinm.2025.103089 40052065 PMC11883405

[56] Larsen N Godt J Grunkin M Lund-Andersen H Larsen M . Automated detection of diabetic retinopathy in a fundus photographic screening population. Invest Ophthalmol Visual Sci. (2003) 44:767–71. doi: 10.1167/iovs.02-0417 12556412

[57] Khansari MM Zhang J Qiao Y Gahm JK Sarabi MS Kashani AH . Automated deformation-based analysis of 3D optical coherence tomography in diabetic retinopathy. IEEE Trans Med Imaging. (2019) 39:236–45. doi: 10.1109/tmi.2019.2924452 31247547 PMC6928449

[58] Wang H Liu X Hu X Xin H Bao H Yang S . Retinal and choroidal microvascular characterization and density changes in different stages of diabetic retinopathy eyes. Front Med. (2023) 10:1186098. doi: 10.3389/fmed.2023.1186098 37564040 PMC10411453

[59] Taylor TR Menten MJ Rueckert D Sivaprasad S Lotery AJ . The role of the retinal vasculature in age-related macular degeneration: a spotlight on OCTA. Eye. (2024) 38:442–9. doi: 10.1038/s41433-023-02721-7 37673970 PMC10858204

[60] Huang Y Rao S Sun X Liu J . Advances in molecular epidemiology of diabetic retinopathy: from genomics to gut microbiomics. Mol Biol Rep. (2025) 52:304. doi: 10.1007/s11033-025-10383-9 40080283

[61] Tavakoli K Huang BB Mirmira T Ma N Weinreb RN Baxter SL . Multi-ancestry genome-wide association study in all of us for primary open-angle glaucoma. Sci Rep. (2026) 16:13788. doi: 10.1038/s41598-026-43993-9 41844886 PMC13129092

[62] Vaswani A Shazeer N Parmar N Uszkoreit J Jones L Gomez AN . Attention is all you need. Adv Neural Inf Process Syst. (2017) 30:5998–6008. doi: 10.65215/r5bs2d54

[63] Dosovitskiy A Beyer L Kolesnikov A Weissenborn D Zhai X Unterthiner T . An image is worth 16x16 words: Transformers for image recognition at scale. ArXiv Preprint ArXiv:201011929. (2020). doi: 10.48550/arXiv.2010.11929

[64] LeCun Y Bottou L Bengio Y Haffner P . Gradient-based learning applied to document recognition. Proc IEEE. (1998) 86:2278–324. doi: 10.1109/5.72679142647864

[65] Goodfellow IJ Pouget-Abadie J Mirza M Xu B Warde-Farley D Ozair S . Generative adversarial nets. Adv Neural Inf Process Syst. (2014) 27:139–44. doi: 10.1145/3422622

[66] Cortes C Vapnik V . Support-vector networks. Mach Learn. (1995) 20:273–97. doi: 10.1007/bf0099401830311153

[67] LaValley MP . Logistic regression. Circulation. (2008) 117:2395–9. doi: 10.1161/circulationaha.106.682658 18458181

[68] Szegedy C Liu W Jia Y Sermanet P Reed S Anguelov D . (2015). “ Going deeper with convolutions”, in: Proceedings of the IEEE Conference on Computer Vision and Pattern Recognition, (Piscataway, New Jersey: IEEE), p. 1–9.

[69] Huang G Liu Z Van Der Maaten L Weinberger KQ . (2017). Densely connected convolutional networks. In: Proceedings of the IEEE Conference on Computer Vision and Pattern Recognition (Piscataway, New Jersey: IEEE) p. 4700–8.

[70] Tan M Le Q . (2019). “ Efficientnet: Rethinking model scaling for convolutional neural networks”, in: International Conference on Machine Learning (ICML) (Cambridge, Massachusetts: PMLR), 6105–14.

[71] Breiman L . Random forests. Mach Learn. (2001) 45:5–32. doi: 10.1023/a:101093340432441886696

[72] Howard AG Zhu M Chen B Kalenichenko D Wang W Weyand T . Mobilenets: Efficient convolutional neural networks for mobile vision applications. ArXiv Preprint ArXiv:170404861. (2017). doi: 10.48550/arXiv.1704.04861

[73] Stone CJ Friedman J Breiman L Olshen R . Classification and regression trees. Wadsworth Int Group. (1984) 8:452–6. doi: 10.1201/9781315139470

[74] Guo T Yang J Yu Q . Diabetic retinopathy lesion segmentation using deep multi-scale framework. BioMed Signal Process Control. (2024) 88:105050. doi: 10.1016/j.bspc.2023.10505042574925

[75] Shaban M Ogur Z Mahmoud A Switala A Shalaby A Abu Khalifeh H . A convolutional neural network for the screening and staging of diabetic retinopathy. PloS One. (2020) 15:e0233514. doi: 10.1371/journal.pone.0233514 32569310 PMC7307769

[76] Akça S Garip Z Ekinci E Atban F . Automated classification of choroidal neovascularization, diabetic macular edema, and drusen from retinal oct images using vision transformers: a comparative study. Lasers Med Sci. (2024) 39:140. doi: 10.1007/s10103-024-04089-w PMC11128386 38797751

[77] Asma K Yassin O Cyrine L Racem C Saker B Afef M . Comparative analysis of OCT-angiography and fluorescein angiography in imaging diabetic retinopathy: Unveiling new diagnostic insights. Eur J Ophthalmol. (2026) 36:58–66. doi: 10.1177/11206721251367571 40791008

[78] Yi G Li Z Sun Y Ma X Wang Z Chen J . Integration of multi-omics transcriptome-wide analysis for the identification of novel therapeutic drug targets in diabetic retinopathy. J Transl Med. (2024) 22:1146. doi: 10.1186/s12967-024-05856-7 39719581 PMC11667901

[79] Silahtaroğlu G Doğuç Ö Furuncuoglu Y . A machine learning model to predict HbA1c values of patients. Bakirkoy Tip Dergisi. (2025) 21:357. doi: 10.4274/BMJ.galenos.2025.2024.4-10

[80] Hannan A Mahmood Z Qureshi R Ali H . Enhancing diabetic retinopathy classification accuracy through dual-attention mechanism in deep learning. Comput Methods Biomechanics Biomed Engineering: Imaging Visualization. (2025) 13:2539079. doi: 10.1080/21681163.2025.253907937339054

[81] Yang J Qin H Por LY Shaikh ZA Alfarraj O Tolba A . Optimizing diabetic retinopathy detection with inception-V4 and dynamic version of snow leopard optimization algorithm. BioMed Signal Process Control. (2024) 96:106501. doi: 10.1016/j.bspc.2024.10650142574925

[82] Tuyet VTH Binh NT Tin DT . A deep bottleneck U-Net combined with saliency map for classifying diabetic retinopathy in fundus images. Int J Online Biomed Eng. (2022) 18:105–22. doi: 10.3991/ijoe.v18i02.27605

[83] Husvogt L Yaghy A Camacho A Lam K Schottenhamml J Ploner SB . Ensembling U-Nets for microaneurysm segmentation in optical coherence tomography angiography in patients with diabetic retinopathy. Sci Rep. (2024) 14:21520. doi: 10.1038/s41598-024-72375-2 39277636 PMC11401926

[84] Gencer K Gencer G Ceran TH Bilir AE Doğan M . Photodiagnosis with deep learning: A GAN and autoencoder-based approach for diabetic retinopathy detection. Photodiagn Photodyn Ther. (2025) 53:104552. doi: 10.1016/j.pdpdt.2025.104552 40064432

[85] Li J Guan Z Wang J Cheung CY Zheng Y Lim L-L . Integrated image-based deep learning and language models for primary diabetes care. Nat Med. (2024) 30:2886–96. doi: 10.1016/j.diabres.2025.112810 39030266 PMC11485246

[86] Wong TY Tham YC Guan Z Li J Cheung C Zheng Y . An integrated image-based deep learning and language models for diabetic retinopathy: a multi-stage development, testing and prospective comparative study. Invest Ophthalmol Visual Sci. (2024) 65:4925.

[87] Mauricio D Gratacòs M Franch-Nadal J . Diabetic microvascular disease in non-classical beds: the hidden impact beyond the retina, the kidney, and the peripheral nerves. Cardiovasc Diabetol. (2023) 22:314. doi: 10.1186/s12933-023-02056-3 37968679 PMC10652502

[88] Wasser LM Liang H-W Li C Cassidy J Tallapaneni P Osterhoudt H . Identifying transportation needs in ophthalmology clinic notes using natural language processing: retrospective, cross-sectional study. JMIR Med Inf. (2025) 13:e69216. doi: 10.2196/69216 40913248 PMC12413321

[89] Fenner BJ Tan T-E Barathi AV Tun SBB Yeo SW Tsai AS . Gene-based therapeutics for inherited retinal diseases. Front Genet. (2022) 12:794805. doi: 10.3389/fgene.2021.794805 35069693 PMC8782148

[90] Li LY Thambawita V Byberg S Hulman A . Assessing the generalisability of foundation models to ultra-wide field retinal imaging for diabetic retinopathy screening in Denmark and Greenland. Int J Med Inf. (2026) 217:106503. doi: 10.1016/j.ijmedinf.2026.106503 42184619

[91] Wu J-H Liu TA Hsu W-T Ho JH-C Lee C-C . Performance and limitation of machine learning algorithms for diabetic retinopathy screening: meta-analysis. J Med Internet Res. (2021) 23:e23863. doi: 10.2196/23863 34407500 PMC8406115

[92] Shah P Farah HA Wisotsky DJ Nawani P Hariharan S Satasia A . Classification of retinal diseases based on optical coherence tomography angiography using cross-modal transfer learning of domain-specific foundation ai models. Br J Ophthalmol. (2026). doi: 10.1136/bjo-2025-329249 42086316

[93] Samanta AK Goyal H Joshi V Mungle T Mitra P . Beyond CLIP: Knowledge-enhanced multimodal transformers for cross-modal alignment in diabetic retinopathy diagnosis. ArXiv Preprint ArXiv:251219663. (2025). doi: 10.48550/arXiv.2512.19663

[94] Pang Y Luo C Zhang Q Zhang X Liao N Ji Y . Multi-omics integration with machine learning identified early diabetic retinopathy, diabetic macula edema and anti-VEGF treatment response. Trans Vision Sci Technol. (2024) 13:23. doi: 10.1167/tvst.13.12.23 39671223 PMC11645727

[95] Li D Huang R Zhou L Tian J Guo S Zou B . Predicting biomolecular interactions via a dual-stream graph neural network with motif constraint and diffusion-based regularization. Comput Biol Chem. (2026) 124:109056. doi: 10.1016/j.compbiolchem.2026.109056 42019100

[96] Thulaseedharan A V I Das DV Jabbar P Gomez R Nair A . Diagnostic accuracy of portable, non-contact, handheld retinal camera for diabetic retinopathy screening. Int J Diabetes Developing Countries. (2026) 46:151–63. doi: 10.1007/s13410-025-01469-y30311153

[97] Sharma S Sharma KP Saini K . (2025). “ A comparative analysis of traditional AI and explainable AI (XAI) for diabetic retinopathy detection: A review”, in: 2025 International Conference on Emerging Technologies and Innovation for Sustainability (Piscataway, New Jersey: IEEE), 116–20.

